# Atomate2: modular workflows for materials science

**DOI:** 10.1039/d5dd00019j

**Published:** 2025-07-01

**Authors:** Alex M. Ganose, Hrushikesh Sahasrabuddhe, Mark Asta, Kevin Beck, Tathagata Biswas, Alexander Bonkowski, Joana Bustamante, Xin Chen, Yuan Chiang, Daryl C. Chrzan, Jacob Clary, Orion A. Cohen, Christina Ertural, Max C. Gallant, Janine George, Sophie Gerits, Rhys E. A. Goodall, Rishabh D. Guha, Geoffroy Hautier, Matthew Horton, T. J. Inizan, Aaron D. Kaplan, Ryan S. Kingsbury, Matthew C. Kuner, Bryant Li, Xavier Linn, Matthew J. McDermott, Rohith Srinivaas Mohanakrishnan, Aakash N. Naik, Jeffrey B. Neaton, Shehan M. Parmar, Kristin A. Persson, Guido Petretto, Thomas A. R. Purcell, Francesco Ricci, Benjamin Rich, Janosh Riebesell, Gian-Marco Rignanese, Andrew S. Rosen, Matthias Scheffler, Jonathan Schmidt, Jimmy-Xuan Shen, Andrei Sobolev, Ravishankar Sundararaman, Cooper Tezak, Victor Trinquet, Joel B. Varley, Derek Vigil-Fowler, Duo Wang, David Waroquiers, Mingjian Wen, Han Yang, Hui Zheng, Jiongzhi Zheng, Zhuoying Zhu, Anubhav Jain

**Affiliations:** a Department of Chemistry, Imperial College London London W12 0BZ UK a.ganose@imperial.ac.uk; b Department of Materials Science and Engineering, University of California Berkeley California 94720 USA; c Energy Technologies Area, Lawrence Berkeley National Laboratory Berkeley CA 94720 USA ajain@lbl.gov; d Materials Science Division, Lawrence Berkeley National Laboratory Berkeley California 94720 USA; e Program in Applied Mathematics, University of Arizona 617 N. Santa Rita Tucson Arizona 85721 USA; f UCLouvain, Institut de la Matière Condensée et des Nanosciences, Université catholique de Louvain Chemin des Étoiles 8, Louvain-la-Neuve 1348 Belgium; g Institute of Physical Chemistry, RWTH Aachen University 52074 Aachen Germany; h Department Materials Chemistry, Federal Institute for Materials Research and Testing (BAM) Berlin Germany; i National Renewable Energy Laboratory Golden Colorado 80401 USA; j Department of Materials, ETH Zürich Zürich CH-8093 Switzerland; k Institute of Condensed Matter Theory and Solid-State Optics, Friedrich Schiller University Jena Jena Germany; l Department of Chemical and Biological Engineering, University of Colorado Boulder Boulder Colorado 80302 USA; m Radical AI, Inc. New York City NY USA; n Thayer School of Engineering, Dartmouth College Hanover NH 03755 USA; o Microsoft Research AI for Science USA; p Department of Civil and Environmental Engineering and the Andlinger Center for Energy and the Environment, Princeton University Princeton NJ USA; q College of Chemistry, University of California Berkeley CA 94720 USA; r Department of Physics, University of California Berkeley CA 94720 USA; s Kavli Energy NanoSciences Institute at Berkeley Berkeley CA USA; t Matgenix SRL, A6K Advanced Engineering Centre Square des Martyrs 1 6000 Charleroi Belgium; u Department of Chemistry and Biochemistry, University of Arizona 1306 East University Boulevard Tucson Arizona 85721 USA; v The NOMAD Laboratory, Fritz-Haber-Institut der Max-Planck-Gesellschaft Faradayweg 4–6 D-14195 Berlin Germany; w Department of Chemistry, University of Colorado Boulder Boulder Colorado 80302 USA; x Cavendish Laboratory, University of Cambridge J. J. Thomson Ave Cambridge UK; y WEL Research Institute Avenue Pasteur 6 1300 Wavre Belgium; z Department of Chemical and Biological Engineering, Princeton University Princeton NJ USA; a Lawrence Livermore National Laboratory Livermore CA 94551 USA; b Molecular Simulations from First Principles – MS1P e.V. Clayallee 167 14195 Berlin Germany; c Department of Materials Science of Engineering, Rensselaer Polytechnic Institute Troy New York USA; d Institute of Fundamental and Frontier Sciences, University of Electronic Science and Technology of China Chengdu 610054 China; e School of Chemistry and Biochemistry, Georgia Institute of Technology Atlanta GA 30332 USA; f Bakar Institute of Digital Materials for the Planet, University of California Berkeley CA 94720 USA

## Abstract

High-throughput density functional theory (DFT) calculations have become a vital element of computational materials science, enabling materials screening, property database generation, and training of “universal” machine learning models. While several software frameworks have emerged to support these computational efforts, new developments such as machine learned force fields have increased demands for more flexible and programmable workflow solutions. This manuscript introduces atomate2, a comprehensive evolution of our original atomate framework, designed to address existing limitations in computational materials research infrastructure. Key features include the support for multiple electronic structure packages and interoperability between them, along with generalizable workflows that can be written in an abstract form irrespective of the DFT package or machine learning force field used within them. Our hope is that atomate2's improved usability and extensibility can reduce technical barriers for high-throughput research workflows and facilitate the rapid adoption of emerging methods in computational material science.

## Abstract 2

We present atomate2, a composable and interoperable workflow engine that extends its predecessor by leveraging the jobflow library and supporting a wide range of calculators (DFT and MLIPs) for dynamic, high-throughput workflow orchestration.

## Graphical abstract

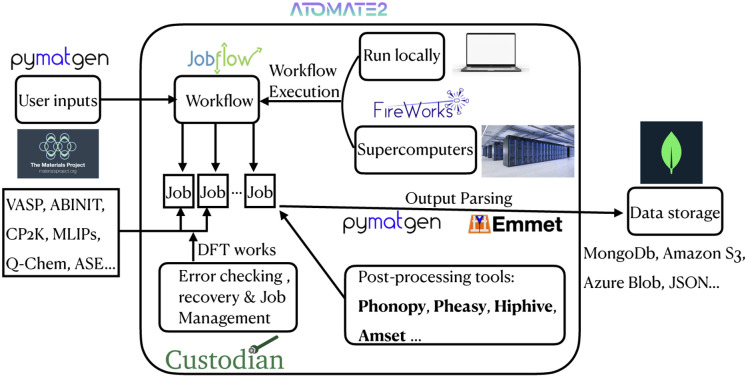

## 1 Introduction

Over the past decade, high-throughput (HT) density functional theory (DFT) calculations have become increasingly popular to the point where they now represent a standard tool within computational materials science. Such calculations serve multiple roles: they enable the screening of materials with specific targeted properties for materials discovery campaigns, enable the development of general-purpose databases of materials properties, and provide foundational data for training machine learning models.

Deploying HT calculations in a generic manner requires a robust software infrastructure. In response to this need, a variety of software frameworks, including AFLOW,^1^ AiiDA,^2–5^ Atomic Simulation Environment (ASE^6^), pyiron,^7^ qmpy,^8^ and our previously developed atomate,^9^ have been developed. Such frameworks have not only made it possible to run DFT calculations at an unprecedented scale, but have also as a side effect made such calculations much more accessible to a larger audience. This is because full automation necessitates the development of automatic parameter decisions, automatic error detection and recovery, and automated execution on heterogeneous computing resources. Such advancements have ultimately resulted in more user-friendly programming interfaces to complex materials calculation procedures.

In this manuscript, we introduce atomate2, an evolution of our earlier work with atomate. atomate2 is designed to enhance the programmability of computational workflows, offer greater flexibility with respect to different simulation models (including those based on MLIPs), support various workflow execution engines, and accommodate a broader spectrum of materials properties with less re-coding. atomate2 represents a comprehensive overhaul of atomate, building on its predecessor's successful application in numerous materials design projects and its integral role in the Materials Project (MP)^10^ database. In the following sections we detail the enhancements and capabilities of atomate2, emphasizing its improved usability and flexibility, which we anticipate will significantly benefit the next wave of HT DFT calculations.

## 2 Atomate2 design philosophy and overview

atomate2 has been designed with the following principles in mind: standardization of inputs and outputs, interoperability between computational methods, and composability of workflows. These goals were informed based on the development and extended usage of the original atomate. Previously, there was not a consistent approach to modify the key parameters of workflows such as inputs and calculation settings. This meant changing default parameters was often an involved process that required the user to inspect the source code for each workflow they intended to run. In atomate2, consistency is enforced by design. For example, all workflows that run using the Vienna *ab initio* Simulation Package (VASP)^11–14^ have the same base set of common options. Changing calculation parameters such as the exchange-correlation functional, modifying the approach used to execute VASP, or writing additional files to the calculation directory, can all be achieved in the same manner irrespective of the specific workflow being performed. This standardization enables workflows to be modified more easily and leads to a more streamlined user experience.

There exists a wide range of DFT packages, each with their own strengths and set of unique features. The atomate package was centered around the use of VASP for periodic systems and Q-Chem^15^ for molecular systems. In atomate2, we have expanded support to a wider array of computational methods including FHI-aims,^16^ ABINIT,^17–20^ and CP2K,^21,22^ in addition to many state-of-the-art machine learning interatomic potentials (MLIPs). Throughout this paper these methods and codes are termed Calculators. A key challenge is to enable heterogeneous workflows where different parts of a workflow are performed using different computational methods. Such workflows are necessary to take advantage of the range of features implemented in different DFT packages. For example, hybrid DFT calculations in CP2K can be significantly accelerated by the auxiliary density matrix method (ADMM), but this implementation is currently limited to the use of a single *k*-point in reciprocal space. atomate2 enables chaining an initial fast hybrid relaxation using CP2K with a slower second relaxation using VASP with denser *k*-point sampling for improved accuracy. Together this simulation procedure can significantly accelerate the computation of complex structures and is a key feature of the heterogeneous defect calculation workflow in atomate2. Achieving interoperability between multiple DFT packages and MLIPs is facilitated by the standardization of workflow inputs and outputs through use of a common application programming interface (API).

Together, standardization and interoperability enable composable workflows. This is a unique feature of atomate2 whereby the substituent parts of a workflow can be seamlessly substituted without impacting the overall workflow execution. This has been facilitated through the use of the jobflow^23^ workflow engine explicitly designed to support “nested” workflows. One example of composability is given by generalizable workflows. For example, the calculation of elastic constants requires obtaining the energy and stress of a series of strained cells before the results are compiled and elastic properties extracted. In atomate2, the elastic constant workflow is defined in an abstract form, where the various parts of the workflow are linked together independent of the computational method used to obtain energies and stresses. The implementation of the workflow for a specific Calculator is as simple as defining the method for a static calculation using that Calculator. The rest of the workflow remains unchanged. Another aspect of composability is the ability to modify workflows in non-trivial ways. For example, the default workflow for point defect in atomate2 is designed to use a single calculation to relax the defect geometry. This calculation can easily be replaced by a sub-workflow that first runs CP2K and then runs VASP as described in the previous paragraph. Again, the workflow definition remains unchanged and is agnostic to the specific sequence of steps, provided the final calculation yields a relaxed structure and the associated energy.

Another aspect of composability is defined by workflow optimization. For example, the FHI-AIMS calculator facilitates the creation of automatic convergence workflows, atomate2 contains a code-agnostic job that performs a series of consecutive code runs with changing inputs, until the absolute difference between the selected result values in two subsequent runs becomes smaller than a predefined value. This job has been used to achieve the *k*-point convergence of energy in static point calculations, as well as the band gap value convergence within the GW framework with respect to the number of frequency points, basis set size, and *k*-point grid used for the self-energy calculation.

Beyond these considerations, atomate2 aims to address several challenges faced by users of the original atomate code. Workflows are written using the jobflow library rather than the FireWorks code, which provides a streamlined experience for complex workflows with modular components. atomate2 broadens the range of databases available for storing large files and objects, including MongoDB (primary document store), Amazon S3, and Azure Blobs, along with simple configuration options for selecting the storage location of specific objects. A high-level summary of the differences between atomate2 and the original atomate is provided in Table 1.

**Table 1:** Overview of key software differences between atomate and atomate2

Code	Workflow engine	Workflow executor	Database	Dynamic workflows	Generalisable workflows	Calculators
atomate	FireWorks	FireWorks	MongoDB	✓	✗	VASP, Q-Chem
atomate2	jobflow	jobflow, FireWorks, jobflow-remote	MongoDB, Amazon S3, Azure Blob, File Storage	✓	✓	Many^a^

a A full list of calculators supported by atomate2 is provided in Table 2.

The computational efficiency and scalability of atomate2 are comparable to those of the original atomate and other modern high-throughput frameworks. In atomate2, as in the original atomate, the heavy-lifting is done by the underlying electronic structure codes (*e.g.*, VASP), so the workflow layer adds minimal runtime overhead. Like other frameworks (*e.g.*, AiiDA or pyiron), atomate2 can fully leverage large-scale computing resources through its workflow execution framework (FireWorks), so it is capable of orchestrating thousands of calculations in parallel with similar efficiency and scalability.

It must be noted that atomate2 differs from ASE in many ways. The writing of input files and parsing of output files is handled primarily by pymatgen (not ASE) in atomate2, specifically for VASP, FHI-aims, ABINIT, CP2K, and LOBSTER. The “orchestration” of a single electronic structure task is executed using bespoke code in atomate2, and involves writing input files, running the external code, and then parsing the results of the calculation into a remote database. For VASP and Q-Chem, additional calls to the custodian python package allow for handling issues that may arise during the calculation. Thus, atomate2 is a framework for these various pieces which can be used to define complex computational workflows in high throughput.

By contrast, ASE is currently used as a backend only for tight-binding Hamiltonian and machine learning forcefield calculations in atomate2. However, its optimization classes are extensible to any ASE calculator, allowing for a high degree of user customization. Again, the actual task orchestration, which involves parsing calculation results into structured, JSON-format output and inserting them into a remote database, is handled by atomate2.

A clear example of this distinction is the MPMorph workflow, which can optionally use a machine learning forcefield to drive its adaptive equation of state fitting of a non-crystalline material. If such a forcefield is used, then ASE is called to perform NVT molecular dynamics at a given volume. However, the determination of which volumes to use, how the range of fitted volumes should be adjusted dynamically, and the parsing and fitting of molecular dynamics runs is all handled by atomate2.

**Fig. 1:**
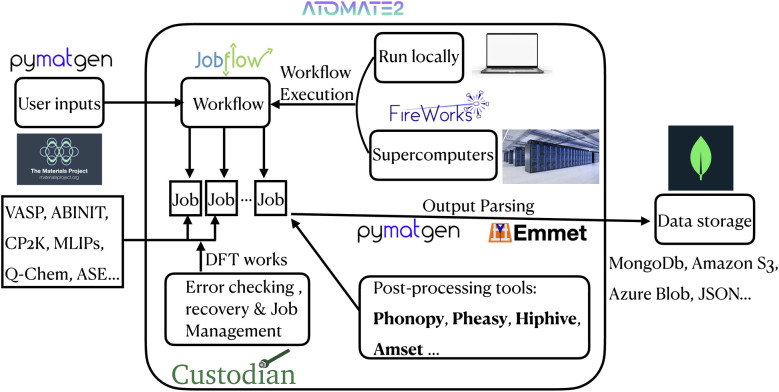
High-level architecture flowchart of the atomate2 framework.

A high-level flowchart that unifies the overall atomate2 architecture into a single diagram can be found in Fig. 1. This flowchart maps the end-to-end data flow. The user structural inputs (*via* pymatgen/Materials Project) feed into the jobflow “Workflow” layer, which decomposes the task into individual jobs dispatched through FireWorks (locally or on supercomputers) with custodian handling error checking and recovery. Upon job completion, raw outputs are parsed by pymatgen and emmet and optionally post-processed by tools such as phonopy, Pheasy, hiPhive, or AMSET before being archived in the user-specified backend (MongoDB, Amazon S3, Azure Blob, JSON, *etc.*). By making the full process explicit, this diagram provides context for each of the detailed workflow diagrams presented later in the paper.

## 3 Calculators

All the supported calculators are mentioned in Table 2.

**Table 2:** Calculators supported by atomate2

Calculators	Periodic system	Non-periodic system
ASE	✓	✓
FHI-AIMS	✓	✓
OpenMM	✓	✓
ABINIT	✓	—
CP2K	✓	—
JDFTx	✓	—
MLIPs^a^	✓	—
VASP	✓	—
Q-Chem	—	✓

a MLIPs include: CHGNet, M3GNet, MACE, GAP, NEP and NequIP.

### 3.1 Atomic simulation environment

ASE is a widely used python package that permits the easy setup of atomistic simulations. ASE simulations are driven by a Calculator class that, given a set of atoms and their positions in 3D space, returns energies and possibly interatomic forces and stresses. This permits structural and molecular relaxation, molecular dynamics (MD), and transition state finding. Abstract ASE workflows for geometry optimization and MD have already been added to atomate2; transition state finding *via* nudged elastic band (NEB)^24^ is currently being added. We use “abstract” here to mean that the workflows require the user to define which ASE-calculator drives the workflow. As examples of how to do this, atomate2 includes concrete implementations of abstract ASE workflows using the Lennard-Jones 6-12 (ref. 25) and GFN*n*-xTB tight-binding Hamiltonian.^26–28^ Note that while ASE is not a Calculator itself, it interfaces with many electronic structure codes, including some directly supported by atomate2.

To take advantage of the rich library of atomistic simulations supported by ASE, atomate2 implements a generic AseMaker class in atomate2 which allows users to define ASE-dependent jobs *via* a Calculator attribute and a run_ase method. This Maker supports both periodic and non-periodic structures as input. The Calculator attribute can be any ASE-compliant Calculator. The run_ase method defines what operations are performed on the input atomic configuration, for example, structural or molecular relaxations *via* the AseRelaxMaker class, or MD *via* the AseMDMaker class. As these classes are easily adapted to a given use case, no workflows are currently implemented in the ASE library.

Outputs from ASE are stored in structured documents: the AseStructureTaskDoc for periodic systems and the AseMoleculeTaskDoc for non-periodic systems. Both document classes inherit from existing document schemas in emmet-core. By default, trajectories (more generally, data for each ionic step) are stored in the user's “large-object” database (such as MongoDB's GridFS) if established.

### 3.2 FHI-aims

FHI-aims^16^ is a community driven, all-electron electronic structure code based on numeric atom-centered orbitals. It supports DFT with a wide range of exchange-correlation functionals, correlated methods beyond DFT (*e.g.* RPA and MBPT), and wave-function based correlation methods (*e.g.* MP2 and CC), as well as *ab initio* MD. It enables first-principles simulations with very high numerical accuracy for production calculations, with excellent scalability up to very large system sizes (tens of thousands of atoms) and up to very large, massively parallel supercomputers. While FHI-aims can treat isolated molecules, clusters, surfaces, and solids on the same footing, it only has atomate2 support for periodic workflows so far. The rest of the section describes the implementation details for the base FHI-aims calculations, and highlights technical details for some workflows.

Currently, the FHI-aims interface to atomate2 can perform both single point and geometry optimization calculations, as well as more complicated workflows. It is also integrated into the phonon, elastic constants, equation of state, magnetic ordering (*via* Mulliken analysis), anharmonicity quantification, and MD workflows of atomate2. All of these provides a template for integrating FHI-aims into other common workflows such as the anharmonic and quasiharmonic phonons. For applications where symmetry is important, this can be activated by using the rlsy_refine_structure keyword in FHI-aims. However, this should not be used when performing calculations on displaced geometries.

All keywords needed to run the calculations are passed to atomate2 through the user_parameters argument and kpt_settings, which are python dictionaries. The default relaxation method used is the trust radius method (TRM) with a maximum allowed force of 1 meV Å^−1^. When running multiple related (same material or molecule) calculations, the atomate2 interface allows for both parallel and serial execution of this job *via* the run_aims and run_aims_socket functions, respectively. The advantage of using the run_aims_socket function is that it uses the i-PI interface^29^ in FHI-aims and the ASE SocketIOCalculator to initialize the electron density to the converged value from the previous calculation when possible, reducing the total number of SCF iterations by a factor of two.

FHI-aims can run the GW calculation in a single shot, without having to restart the calculation after completing an SCF cycle. Such a run will consist of an SCF part, during which the ground-state electronic density is obtained, and a post-SCF part when the GW self-energy is evaluated. However, the two parts can also be separated using FHI-aims restart capabilities. The GW workflow for FHI-aims, implemented in the atomate2 package, dumps the resulting SCF eigenfunctions and reads them at the beginning of the GW run. It helps in several ways by: (1) making calculations more computationally efficient, (2) achieving consistency in the results, and (3) allowing more flexible exploration of the parameters space.

### 3.3 ABINIT

ABINIT is an open-source first-principles software implementing a diverse range of formalisms such as DFT, density-functional perturbation theory (DFPT), many-body perturbation theory (GW approximation and Bethe–Salpeter equation), and dynamical mean-field theory among others. Since it relies on plane waves to represent the wavefunctions, periodic boundary conditions are imposed. ABINIT is thus particularly suited to deal with periodic structures, although this limitation can be circumvented by embedding non-periodic systems in the appropriate supercell. Both norm-conserving pseudopotentials and the projector-augmented wave method are supported. Numerous quantities can be calculated including electronic, vibrational, optical, magnetic, mechanical, and thermodynamic properties.

At present, standard DFT tasks, that is, structural relaxation (atoms and/or cells), SCF- and NSCF-calculation (uniform or bandstructure) as well as many-body perturbation theory (MBPT) calculations such as quasiparticle energies within the GW approximation and dielectric function calculation by solving the Bethe–Salpeter equation, are interfaced within atomate2. The plan is to port the previously developed abiflows package, which was among others used to calculate 1521 semiconductors in the harmonic approximation in collaboration with the MP,^30^ to atomate2. In this regard, the abiflows DFPT workflow for calculating the static second-harmonic generation tensor^31^ (and the static dielectric tensor) has been implemented in atomate2 and is under review.

The global machinery heavily relies on functionalities provided by abipy such as the automatic input generation and outputs processing.^20^ Following the philosophy of atomate2, each Maker or calculation type (inheriting from BaseAbinitMaker) has its own AbinitInputGenerator, which in turn calls a specific abipy factory function to generate the proper AbinitInput. Once a job is completed, the parsing capabilities of abipy are fully leveraged to retrieve relevant outputs. Indeed, abipy provides a specific parser class for each file, whether text or netcdf. The available methods of those parsers allow to construct an AbinitTaskDoc following the same schema as for the other codes with common basic fields such as output.energy, output.bandgap or output.forces. In addition, it is possible to directly store relevant files such as the DDB or netcdf ones into a FileStore partition of the interacting MongoDB. They can then be retrieved at will for further manipulation with abipy such as automatic plots generation of bandstructure, density of state, or spectra. When possible, basic figures are already saved to allow a quick inspection. By default, the ABINIT workflows will look for pseudopotentials from the Pseudodojo^32^ in the default folder (/.abinit/pseudos). It is thus necessary to download them using the abipy abips.py command. The –help option lists the valid subcommands such as avail, list, and install. Although difficult, it is possible to use custom pseudopotentials. Active developments are focusing on improving this aspect.

### 3.4 CP2K

CP2K is an open-source software package for performing atomistic simulations including electronic structure calculations using DFT and (post) Hartree–Fock (HF) methods as well as MD simulations using classical force fields. CP2K uses analytic Gaussian-type orbitals to form a local basis set, which can be used to simulate both periodic and non-periodic systems. Additional unique features include the auxiliary density matrix method (ADMM) for accelerating hybrid DFT calculations, the Gaussian and Augmented Plane Waves (GAPW) method for scalable all-electron calculations, and linear scaling DFT methods. Basic DFT tasks with CP2K^21,22^ have been interfaced with atomate2 using jobflow.

### 3.5 JDFTx

JDFTx^33^ is an open-source plane-wave DFT code that supports grand canonical DFT (GC-DFT) and implements advanced implicit solvent models. GC-DFT and JDFTx are particularly useful for studying solvated interfaces that are relevant in electrochemical applications. DFT and GC-DFT structure optimization jobs with and without solvent are supported in atomate2. Default solvation and DFT parameters are set in accordance with the BEAST Database,^34^ a database of electrocatalysis GC-DFT data hosted by the National Renewable Energy Laboratory.

JDFTx uses the GC-SCF and AuxH electronic algorithms, which outperform outer-loop grand canonical electronic algorithms found in other codes.^35^ Advanced solvation models are available including the non-linear non-local SaLSA implicit solvent model as well as CANDLE, a linear implicit solvent model with asymmetric charge response.^36,37^ JDFTx can also be integrated directly into excited state calculations in BerkeleyGW,^38^ although atomate2 support for GW workflows with JDFTx is not expected soon. JDFTx output and input files are parsed with code in pymatgen.io.jdftx. JDFTx log files, eigenvalue and bandProjection files are currently supported by the parsers.

### 3.6 Force fields

MLIPs have become increasingly useful to computational materials scientists. At the time of writing, several modern MLIPs have atomate2 interfaces including MACE-MP-0,^39^ CHGNet,^40^ M3GNet,^41^ NEP,^42–44^ NequIP,^45^ SevenNet,^46^ and GAP.^47,48^ MLIPs are currently accessible in atomate2 *via* ASE Calculators and the infrastructure of the Section 3.1. These models can be used either directly as Calculators or can be incorporated into hybrid workflows that use the MLIPs to pre-relax a structure and then feed it into a DFT relaxation in VASP. This MLIP pre-relaxation can be implemented within several other DFT-based workflows to reduce computational cost.

Moreover, fully MLIP-based workflows have been implemented as well. Specifically, one can use any of the supported MLIPs to calculate the elastic constant tensor or harmonic phonons of a material as recently demonstrated with MACE-MP-0. Other applications of the force field Calculators and workflows include ref. 49. Substituting DFT-based Calculators with MLIPs allows faster and cheaper runs, and makes atomate2 an ideal tool for easily reproducible benchmarking against DFT calculations. More details on the respective implementations can be found in the corresponding workflow sections below. Additionally, MLIP molecular dynamics (MLMD) calculations have been incorporated for the micro-, grand-, and canonical ensembles, with more complex workflows using MLMD to, *e.g.*, rapidly equilibrate amorphous structures. Virtually all workflows which do not require electronic properties can be adapted to MLIPs, such as quasi-/harmonic phonon calculations and equation of state properties.

### 3.7 VASP

VASP is a licensed, pseudopotential, plane-wave electronic structure code. While VASP primarily performs non-dynamical and *ab initio* MD (AIMD) DFT calculations, it is also capable of performing many-body perturbation theory (MBPT) calculations *via* the random phase approximation, GW approximation, and Bethe–Salpeter equation (BSE). VASP primarily uses projector augmented wave (PAW) pseudopotentials,^50^ but can also use ultrasoft pseudopotentials, both of which are in a proprietary format.

VASP is the main code used by the Materials Project to generate structural, electronic, and thermodynamic materials data, and thus has a wide breadth of workflow coverage. Within atomate2, VASP-based tasks and workflows include: geometry optimization, single-point calculations, AIMD, equation of state, band structure scans, phonon dispersion, amorphous solid equilibration, *etc.* Transition state workflows based on nudged elastic band (NEB)^24^ and ApproxNEB^51^ are currently being added for VASP.

The VASP Calculators in atomate2 rely on pymatgen^52^ to define input sets (minimally, the INCAR, POSCAR, and POTCAR files) which are defined in the pymatgen.io.vasp.sets library. The output of a VASP calculation is parsed by emmet^53^ into its TaskDoc schema. This schema is sufficiently flexible to incorporate key electronic structure information from non-dynamical DFT, AIMD, and MBPT calculations. By default, jobs are run with the custodian package^54^ to monitor for VASP and computational resource errors and possibly correct these on the fly.

In atomate2, VASP input files are represented as JSONable objects *via* the VaspInputGenerator class. This class lightly wraps pymatgen's VaspInputSet class with appropriate defaults set for high-throughput calculations.^52^ These sets essentially determine which kind of calculation is run, for example: geometry optimization, static single-point energy calculation, band structure calculation, or AIMD. A single VASP calculation is represented as a jobflow Maker object, which can then be chained together to form workflows (jobflow Flow objects). At present, nearly all legacy atomate VASP jobs and workflows have been ported to atomate2 and many new workflows have been added.

### 3.8 Q-Chem

Q-Chem is a comprehensive *ab initio* electronic structure software package designed to handle molecular systems. It offers an extensive array of computational methods to enable the calculation of ground and excited states with speed and accuracy. Among its capabilities, Q-Chem supports density functional theory (DFT) with a wide variety of basis sets and functionals, wavefunction-based methods like coupled cluster (CCD, CCSD) calculations, and perturbation techniques such as MP2. Additionally, it accommodates Time-Dependent DFT (TDDFT), ΔSCF methods, and specialized techniques such as restricted and complete active space (RAS and CAS) approaches. These advanced functionalities are invaluable for examining excited states and calculating spectroscopic properties, such as core ionization energies.

The atomate2 Q-Chem integration supports several fundamental tasks, including geometry optimization, single-point energy calculations, and frequency analysis. More sophisticated tasks, like potential energy surface (PES) scans and transition state optimizations, are also available. The interface with pymatgen facilitates these tasks through the InputGenerator and InputSet architecture. This design allows users to encapsulate all calculation settings into a QCInputGenerator class, which, when provided with a molecule from the pymatgen library, produces a complete set of Q-Chem inputs specific to that molecule.

The infrastructure is highly customizable, making it amenable for advanced users to implement new jobs and workflows. The Maker class in the jobflow library forms the backbone for constructing and managing these computational jobs. A key component, the BaseQCMaker, utilizes the QCInputGenerator to yield a QCInputSet from a given pymatgen molecule while supporting additional parameters for job execution, error-handling, and result documentation. Q-Chem calculators within atomate2 automatically archive inputs and outputs using a structured schema known as a Task Document, defined in the emmet.core.qc_tasks TaskDoc class. This schema ensures standardized data processing by storing specific results (*e.g.*, final energy) in predefined attributes (TaskDoc.output.final_energy). The TaskDoc is easily serialized for integration into a results database (*e.g.* MongoDB) or storage as a local JSON file, ready for automated handling by tools such as the Builder classes in Emmet.

## 4 Workflows

The workflows included in atomate2 are based on robust, published methodologies, many of which have been rigorously validated through convergence testing and experimental benchmarking. For workflow-specific validation details, we refer the reader to the original publications listed in the documentation corresponding to each workflow. It is also important to note that the accuracy of properties computed using atomate2 workflows ultimately depends on the underlying calculator (*e.g.*, DFT engine or MLIP). While atomate2 provides robust and reproducible workflow logic, benchmarking of individual codes is performed independently and documented in separate publications.^55–57^ Users are encouraged to refer to these studies when evaluating results produced by new or alternative calculators.

**Fig. 2:**
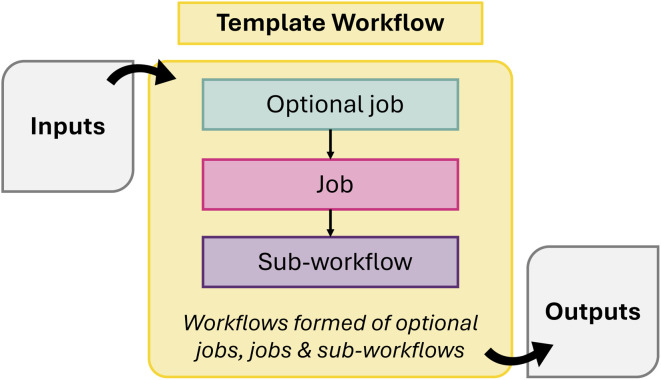
Schematic of template abstract workflow including color legend.

A list of all the supported workflows are listed in Table 3. Each workflow covers the methodology, usage notes, and any existing use cases/papers using the workflow, and has a workflow diagram covering all the steps. A template workflow diagram, providing a legend to enable their interpretation is shown in Fig. 2.

**Table 3:** Workflows and their corresponding supported Calculators. The MLIP column represents all models currently supported by atomate2 including CHGNet, M3GNet, MACE, GAP, and NequIP. ○ indicates supporting this workflow-engine combination is on atomate2's near-term development roadmap. △ is used for combos that are not currently planned but are considered a small implementation effort that could be added by adapting similar existing Makers

Workflow	System	Supported calculators								
		ASE	FHI-AIMS	ABINIT	CP2K	JDFTx	MLIPs	VASP	OpenMM	Q-Chem
Geometry optimization	Both	✓	✓	✓	✓	✓	✓	✓	✓	✓
Static	Both	✓	✓	✓	✓	✓	✓	✓	✓	✓
Dielectric	Periodic	—	—	—	—	—	—	✓	—	—
Polarization	Periodic	—	—	—	—	—	—	✓	—	—
Electronic band structure	Periodic	—	✓	✓	✓	—	—	✓	—	—
Bond analysis workflow with LOBSTER	Periodic	—	—	△	—	—	—	✓	—	—
Excited states (GW-BSE)	Periodic	△	✓	✓	✓	—	—	✓	—	—
*Ab initio* molecular dynamics	Periodic	✓	△	—	—	—	—	✓	—	—
Force field molecular dynamics	Periodic	✓	✓	—	—	—	✓	✓	—	—
Elastic constants	Periodic	△	✓	△	△	—	✓	✓	—	—
Harmonic phonons	Periodic	△	✓	△	△	—	✓	✓	—	—
Equation of state	Periodic	△	✓	△	△	—	✓	✓	—	—
Electron–phonon	Periodic	—	—	—	—	—	—	✓	—	—
Grüneisen	Periodic	—	△	—	—	—	✓	✓	—	—
Matpes	Periodic	—	—	—	—	—	—	✓	—	—
Quasiharmonic approximation for phonons	Periodic	—	△	—	—	—	✓	✓	—	—
Anharmonic phonons	Periodic	△	○	△	△	—	✓	✓	—	—
MPMorph	Periodic	△	△	△	△	—	✓	✓	—	—
Magnetic ordering	Periodic	△	✓	△	△	—	—	✓	—	—
Adsorption	Periodic	△	△	△	△	○	△	✓	—	—
Point defect	Periodic	△	○	—	—	—	—	✓	—	—
Anharmonicity quantification	Periodic	△	✓	△	△	—	△	△	—	—
Electrode discovery	Periodic	△	—	—	—	—	—	✓	—	—
Ferroelectric	Periodic	△	—	—	—	—	—	✓	—	—
Materials project	Periodic	—	—	—	—	—	—	✓	—	—
Amset	Periodic	—	—	—	—	—	—	✓	—	—
Frequency flatterning optimizer workflow	Molecular	—	—	—	—	—	—	—	—	✓
Classical molecular dynamics workflow	Molecular	✓	—	—	—	—	—	—	✓	—

### 4.1 Periodic systems

#### 4.1.1 Geometry optimization and static

As a starting point, atomate2 offers several essential DFT jobs, including structural optimization and single-point (static) calculations. The crystalline structure can be provided in various formats supported by pymatgen, including Crystallographic Information File (CIF), POSCAR, and other commonly used structure file formats. Conveniently, pymatgen offers the get_structure_by_material_id() function, which allows users to query a structure from the Materials Project database using its corresponding mp_id. For structural optimization, both HSE06 (ref. 58) and PBE^59^ regular relaxation and tight relaxation jobs are available. Additionally, atomate2 includes a powerups function, allowing users to customize their input settings. For example, functions like update_user_incar_settings, update_user_kpoints_settings, and update_user_potcar_settings enable tailored configurations for DFT calculations. Similarly, for static calculations aimed at evaluating the total energy of compounds and generating the CHGCAR file for subsequent band structure calculations, atomate2 provides support for both conventional functionals and hybrid functionals. The subsequent sections delve into more advanced workflows, most of which incorporate structure relaxation and static calculations as integral components of their process.

#### 4.1.2 Dielectric and polarization workflow

**Fig. 3:**
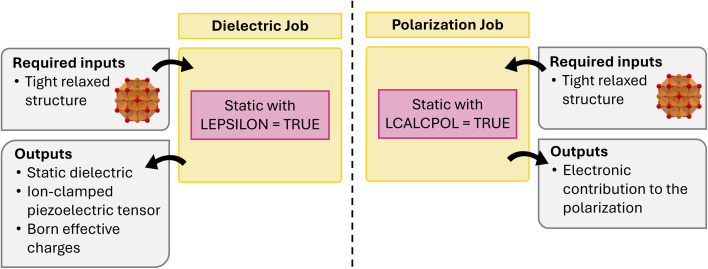
Schematics of dielectric and polarization workflows.

atomate2 also supports other fundamental calculations including dielectric^60^ and polarization jobs. It should be noted that a pre-relaxed structure is required as input for both calculations. This is to avoid imaginary modes. These calculations are currently available for VASP and the workflow is summarized in Fig. 3. The corresponding workflows in atomate have been widely employed, including the generation of over 7000 dielectric tensors in the Materials Project database. This dataset is one of the core components of the MatBench^61^ benchmarking suite for comparing ML models on materials science tasks and has been used to develop equivariant graph neural networks such as AnisoNet^62^ for predicting the full dielectric tensors of crystalline systems.

#### 4.1.3 Electronic bandstructure workflow

**Fig. 4:**
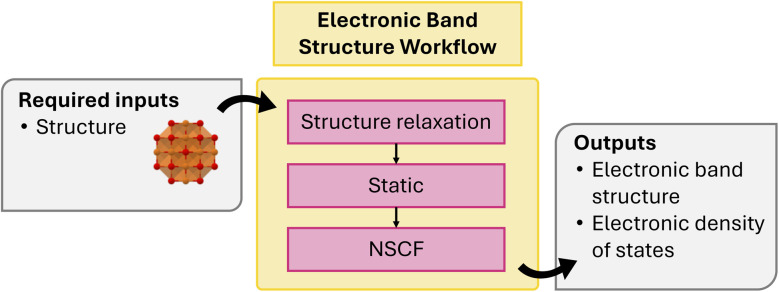
Schematic of electronic bandstructure workflow.

A fundamental and widely utilized application of DFT is the calculation of electronic band structures and density of states (DOS) to characterize the electronic properties of materials. To obtain the electronic band structure and density of states (DOS) in atomate2, the workflow (Fig. 4) begins with a precise structural relaxation, followed by a static calculation to generate the CHGCAR file required for subsequent steps. Next, non-self-consistent static calculations are performed using either *k*-points along a high-symmetry path or a uniform *k*-point mesh.

#### 4.1.4 Bonding analysis workflow with LOBSTER

Bonding analysis helps to understand the interactions between constituent atoms in materials. Theoretical frameworks for bonding analysis usually rely on density-based or quantum-chemical orbital-based approaches. One of the commonly used density-based approaches is the Bader^63^ analysis. Orbital-based approaches typically rely on the Mulliken^64^ population analysis, from which one can further derive the Crystal Orbital Overlap Populations (COOP),^65^ the Crystal Orbital Hamilton Populations (COHP),^66^ and the Crystal Orbital Bond Index (COBI).^67^ The Local-Orbital Basis Suite Towards Electronic-Structure Reconstruction (LOBSTER)^68–70^ software package can perform quantum-chemical orbital-based bonding analysis and can recover COOP, COHP, and COBI populations by projecting plane-wave-based wave functions from modern density functional theory computations onto atomic orbitals.

**Fig. 5:**
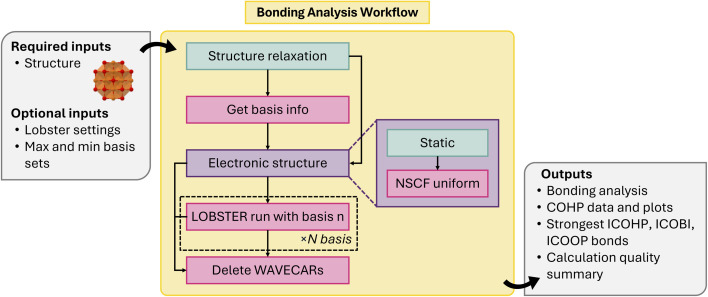
Schematic of bonding analysis workflow.

The workflow (Fig. 5) involves the following steps: (1) structural optimization, (2) calculating the number of bands based on available projection basis functions, (3) writing static calculation inputs with the number of bands set equal to as evaluated in step 2, (4) performing a static self-consistent, plane-wave based DFT calculation with symmetry, (5) performing a static non-self-consistent plane-wave based DFT run with symmetry switched off to compute the wave function, (6) generating a set of LOBSTER computations based on different combinations of available atomic orbital basis functions for projection of the wavefunctions, (7) running LOBSTER computations and analyzing outputs automatically with LobsterPy for each of the LOBSTER runs, (8) deleting the wavefunction files from the static calculation and LOBSTER runs directories.

This workflow originates from a previously implemented workflow in atomate.^71^ The latter was used to produce a database for about 1500 semiconductors and insulators.^72^ The key methodological difference in the previous implementation and workflow in atomate2 is that the wave function is now computed in a two-step procedure including a self-consistent DFT run with symmetry and a non-self consistent DFT run without symmetry to speed up the computation. In addition, now an analysis of the outputs *via* the LobsterPy^71,73^ package is performed. Important to less experienced users of HT software, the atomate2 framework enables efficient workflow execution on one computing node with a simple submission script and only a minimal setup. Only the installation of atomate2 and the configuration of the run commands for VASP and LOBSTER are required. This significantly lowers the barrier to workflow usage in contrast to atomate. Structural optimization and static DFT runs are performed in this workflow using VASP. The automatic analysis *via* LobsterPy is performed for symmetrically inequivalent sites in the structure for cation–anion and all bonds. This analysis involves identifying the most relevant bonds along with coordination environments based on Integrated Crystal Orbital Hamilton Populations (ICOHPs), numerical evaluation of bonding-antibonding contributions, corresponding Crystal Orbital Hamilton Populations (COHP) plots, a JSON summary, and text description of the bonding analysis. More details about our automatic bonding analysis implementation can be found in the publications associated with LobsterPy^71,73^ and its tutorials.

#### 4.1.5 Excited states workflow

DFT, being an exact theory for ground state properties, often works well to compute structural properties but doesn't provide accurate excited state properties such as band gaps. A more rigorous framework for the description of excited states is provided by MBPT^74,75^ based on Green's functions and the concept of quasiparticles. The quasiparticle energies are the energies for adding or subtracting an electron from a many-electron system. Using the same Green's function-based MBPT framework, neutral excitations, which can be directly compared to experimental optical absorption spectra, can also be calculated from the solution of the Bethe–Salpeter equation (BSE).^76,77^ The BSE represents one of the most accurate yet computationally tractable approaches for the *ab initio* study of neutral excitations in crystalline systems by including the attractive interaction between electrons and holes (excitonic effects) using two-particle Green's function thus going beyond the single-particle picture of DFT within random-phase approximation (RPA).

The GW and BSE workflows implemented in atomate2 are built for automating these multistep and interdependent calculations. For example, the calculation of quasiparticle energies using Abinit involves a four-step calculation. First, one performs a standard DFT (SCF) calculation to obtain self-consistent charge density which is then used to perform an exact diagonalization calculation (NSCF) to generate a large number of unoccupied bands required for the actual GW calculation. Once these bands are generated the inverse dielectric matrix is computed (SCR) and used to obtain the quasiparticle corrections to the DFT eigenvalues (SIGMA). Similarly, the BSE calculation involves obtaining the inverse dielectric matrix and quasiparticle corrections to compute the frequency-dependent macroscopic dielectric function (*ε*(*ω*)). Workflows such as G0W0Maker and BSEFlowMaker perform such standard calculations with a given crystal structure and input parameter set. In addition, multiple workflows have been developed to check the convergence of desired output results with any particular input parameter. For example, GWConvergenceMaker implements the convergence test of the calculated quasiparticle gap with respect to parameters such as the number of unoccupied bands or the number of plane waves. The BSEConvergenceMaker checks the convergence of the frequency-dependent dielectric function calculated using BSE with the number of *k*-points. Due to the enormous computational cost of these calculations, a BSEmdfMaker has been developed that performs the BSE calculation with a model dielectric function. One can also use a scissor shift to simulate the quasiparticle correction and skip the SCR and SIGMA jobs mentioned earlier.

The implementation of the GW workflow for FHI-aims is simpler, as FHI-aims allows the user to run a SCF calculation and all the post-SCF steps in one run. However, if one wants to study the convergence of the quasiparticle energies on the parameters defining the GW calculation, such as the number of frequency points used to expand the elements of self-energy on the imaginary frequency axis, or the type of its analytical continuation on the real frequency axis,^78^ they can effectively re-use the results of the SCF part of the calculation by restarting the calculations from the converged charge density. This functionality is implemented in GWMaker for FHI-aims. GWConvergenceMaker, allowing the study of the convergence of GW results with respect to calculation parameters, is also implemented similarly to the ABINIT workflows.

In addition to the GW workflow for FHI-aims, atomate2 also implements the workflow to compute *G*_0_*W*_0_ quasiparticles with VASP in the MVLGWBandStructureMaker class. It conducts *G*_0_*W*_0_ calculations compatible with the parameters defined by MVLGWSet in pymatgen. First, a static DFT calculation is performed using the MVLStaticMaker by default, which can be customized with a user-defined static maker. Next, a non-self-consistent calculation is carried out with the MVLNonSCFMaker, starting from the CHGCAR produced in the static calculation. Finally, the MVLGWMaker class builds the dielectric matrix, performs the *G*_0_*W*_0_ calculations, and obtains the quasiparticle energies.

#### 4.1.6 *Ab initio* and forcefield molecular dynamics workflows

Molecular dynamics (MD) simulations are important for sampling atomistic configurations of systems at finite temperature and pressure, and have been widely used for calculating the thermodynamic responses and properties of materials such as heat capacity, viscosity, and thermal conductivity. *Ab initio* MD (AIMD) generally refers to the use of electronic structure methods (typically DFT) to dynamically update the positions of atoms in a system,^79^ whereas forcefield MD refers to any method that uses a model for interatomic forces to drive the simulation. We will further distinguish classical MD, where the functional form of a forcefield is constructed and fitted by hand, and machine-learned MD, where the forcefield is represented by a trained ML model.

AIMD workflows are available for VASP *via* MDMaker, which allows easy selection of common options like the temperature and the ensemble (*NVE*, *NVT*, *NpT*), with suitable default choices for the thermostat and/or barostat. It is also possible to run AIMD using the ASE calculator interfaces to electronic structure codes, such as Q-Chem, SIESTA, Quantum Espresso, VASP, and others. One could use the AseMaker class in atomate2 to communicate with electronic structure codes and use their native AIMD implementations. Alternatively, one could use the AseMDMaker to take energies, forces, and stresses from a single-point electronic structure calculation, and perform AIMD using ensembles defined internally in ASE.

**Fig. 6:**
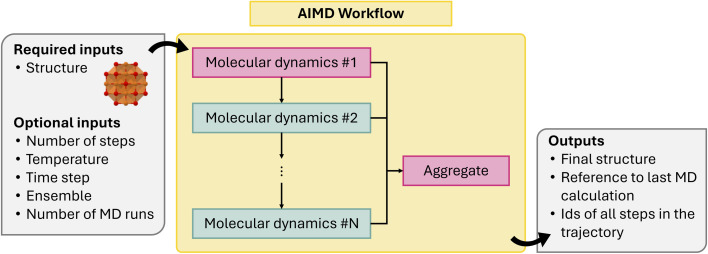
Schematic of multi-step molecular dynamics (MD) workflow.

One of the main challenges in MD simulations is the number of ionic steps that should be performed to extract reliable information from the post-processing of the data. A total simulation time of a few ps or a few tens of ps is usually required, with a time-step in the order of the fs. Considering that simulation boxes often include up to hundreds of atoms, this can be particularly challenging for AIMD, where the total simulation time can easily exceed the maximum time per job allowed by computing centers. To address this issue, a multi-step MD workflow (MultiMDMaker) has been implemented. This permits splitting the total simulation time in a customizable number of chunks so that each separate MD calculation can finish within the allotted time. A final job is added to summarize the output and provide references to the different output chunks so that the total trajectory can easily be reconstructed. In addition, the final document can be used as a starting point for a new MultiMDMaker workflow, enabling the user to concatenate multiple such workflows. This workflow is illustrated in Fig. 6.

The same MultiMDMaker workflow can also be used to concatenate trajectories with different thermo- and barostat profiles, a feature that is not currently implemented in VASP. For example, the workflow can define an initial chunk with a ramp-up of the temperature, followed by additional steps at constant temperature.

MLMD is made possible by the MD ensembles (*NVE*, *NVT*, and *NpT*), thermostats, and barostats defined in the ASE python package. It is also possible to use the native MLIP functionality of VASP to perform MD with the previously-mentioned atomate2.vasp.MDMaker class. To make the ASE interface forward-looking, an AseMDMaker template has been defined, which defines the ensemble and various computational parameters for a generic ASE Calculator object. The AseMDMaker supports both periodic and non-periodic systems. To perform MLMD, users can access pre-defined CHGNet, GAP, M3GNet, MACE-MP-0, and Nequip MD workflows, or they can load any force-field ASE Calculator by specifying the package to import. Both temperature and pressure scheduling features have been added for force field MD Makers. If arrays of temperature and pressure are input, the temperature and pressure will be linearly interpolated across simulation steps, allowing users to customize MD simulations with, *e.g.*, temperature ramp, annealing, or cyclic expansion–compression loading. The highly modular nature of the MLIP MD workflows makes them amenable to inclusion in complex workflows, such as the MPMorph workflows^80,81^ used to simulate quenched amorphous structures. This enables the rapid generation of amorphous structures at a much lower cost than DFT and is being actively explored as an application of MLMD.

**Fig. 7:**
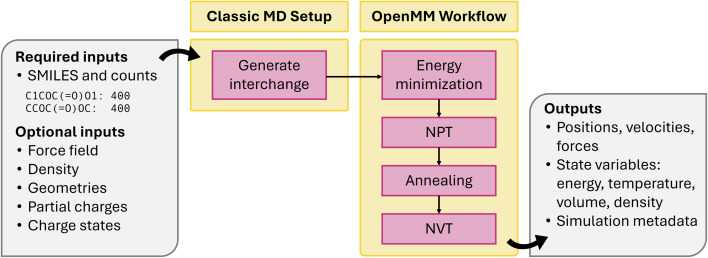
Schematic of classical MD workflow.

Last, classical MD is a popular technique for investigating electrolytes, polymers, proteins, and a wide variety of other systems, particularly when bond breaking and formation is not of interest. This module shown in Fig. 7 includes (1) an extensible MD engine-agnostic schema and setup tools built on the open force field ecosystem and (2) MD workflows for energy minimization, *NpT*, *NVT*, and annealing. Together, these allow facile system construction, atom typing, execution, and analysis. Representing the intermediate state of a classical MD simulation is challenging. While the intermediate representation between stages of a periodic DFT simulation can include just the elements, Cartesian coordinates, and box vectors, classical MD systems must also include the force field. This is particularly challenging because all MD engines represent force fields differently. Rather than implement our own representation, the workflow uses the openff.interchange.Interchange object, which catalogs the necessary system properties and interoperates between several MD engines. Alongside this, the workflow tracks convenient metadata not critical to the simulation, like molecule names and partial charge methods. For system setup, a generate_interchange job in atomate2.classical_md.base has been implemented, which processes a simple input dictionary into a task document. Though the task document is designed to be easily used by multiple MD codes, the existing workflows are in OpenMM. OpenMM workflows are built around the BaseOpenMMMaker, which includes shared logic to create a OpenMM.Simulation, attach OpenMM.Reporters, and output a task document. Jobs subclass BaseOpenMMMaker and implement a unique run_openmm method, which evolves the system as needed. Several Makers are implemented: NVTMaker, NPTMaker, TempChangeMaker, and EnergyMinimizationMaker. A common challenge in HT MD workflows is the selection of equilibration timescales *a priori*.^82,83^ To this end, the DynamicOpenMMFlowMaker is implemented to enable continuous execution of any BaseOpenMMMaker subclass in discrete stages while monitoring physical observables (*e.g.*, potential energy, density, *etc.*) until custom convergence criteria are met. Unlike other codes supported by atomate2, OpenMM is run through a python API and has no notion of input files. Instead of building and writing input sets, the workflow implements simulation logic directly in python.

#### 4.1.7 Elastic constant workflow

The elastic tensor is a fundamental material property that describes the mechanical response of a material to small external loads, offering a complete description of the material's behavior under such conditions. A variety of mechanical, thermal, and acoustic properties can be directly derived from the elastic tensor. Computationally, there exist two major methods to calculate the elastic tensor using first-principles calculations. The first is the energy–strain approach, which relates the elastic tensor to the second derivative of the total energy with respect to strain. The second is the stress–strain approach, where the elastic tensor *C* is obtained by leveraging the linear relationship between stress *σ* and strain *ε*, that is, *σ* = *Cε*. The present workflow implements the stress–strain approach; a detailed explanation of this approach can be found in ref. 84.

The elastic workflow takes an atomic structure as input and produces the elastic tensor as output. This is accomplished through a series of steps detailed below. First, as two optional steps, the input structure can be further optimized and converted to a conventional cell.^85^ Using a conventional cell can help reduce numerical errors, particularly for crystals whose primitive cell can be highly skewed, such as monoclinic and triclinic systems. Next, the structure is strained along the six independent strain directions (*xx*, *yy*, *zz*, *yz*, *xz*, and *xy*), with multiple strain magnitudes applied in each direction to deform the structure. To further optimize the process, optionally, the set of strained structures can be reduced by symmetry, which involves checking if a strained structure is equivalent to another using the space group symmetry operations of the original structure. This can significantly reduce the number of structures to be calculated, particularly for high-symmetry structures like cubic systems. Next, a Calculator is employed to compute the stress tensor for each strained structure, and any atomate2 Calculator that supports stress tensors, as mentioned in the Calculators section, can be used for this computation. Finally, the sets of strains and stresses are used to fit the elastic tensor using the strain–stress relationship, as implemented in pymatgen.

The output is a fourth-rank elastic tensor corresponding to the input structure, with different crystal systems possessing different numbers of independent components according to the symmetry in the crystal. Many other isotropic and anisotropic elastic properties can be directly derived from the elastic tensor, such as Young's modulus, shear modulus, bulk modulus, Poisson's ratio, linear compressibility, and sound velocity. Numerical values of these derived properties can be obtained *via*, *e.g.* pymatgen, and visual exploration is made possible *via* packages like elate.^86^

The present workflow is a direct adaptation of the original elastic workflow in the atomate package. Aside from default settings such as DFT pseudopotentials and energy cutoffs, the main difference is that this workflow includes the option to further optimize the input structure. The original elastic workflow has been widely employed to calculate the elastic tensor of materials.^84,87,88^ Notably, the elastic tensor data provided in the MP database are computed using this workflow. These data are driving the development of modern machine-learning models for predicting the elastic properties of materials. For instance, derived mechanical properties such as bulk modulus and shear modulus serve as key benchmarking properties in the MatBench suite.^61^ Furthermore, the data have been utilized to develop equivariant graph neural networks such as MatTen^88^ for predicting the full elastic tensors of all crystal systems.

#### 4.1.8 Harmonic phonons workflow with phonopy

Lattice dynamics govern thermal conductivity, phonon transport, heat capacity, and other mechanical, optical, and electrical properties. High-accuracy phonon dispersion relations are essential for understanding these relationships. The finite displacement approach is one of the most widely used methods to obtain phonon dispersions, mostly because it is applicable to any atomistic force Calculator. Density functional perturbation theory, in contrast, needs to be derived and implemented for each DFT functional. The finite displacement-based computation is time-consuming as it requires the calculation (and collection) of all interatomic forces for a large number of supercells.

Using phonopy^89,90^ as the underlying framework, the atomate2 implementation of harmonic phonon workflow requires forces that can be computed from DFT calculations (VASP or FHI-aims), but also from (universal) MLIPs (*e.g.*, M3GNet, CHGNet, MACE-MP-0, NEP, NequIP). phonopy handles the creation of supercells with single displacements and subsequent calculation of the dynamical matrix. Based on DFT calculations, the non-analytical term correction^91^ (NAC) can be included in the workflow to account for polarisation effects on the force constants near *Γ* in non-metallic systems. This can be combined with both DFT or MLIP Calculators. Unfortunately, current MLIPs model atomic structure only and have no notion of electronic degrees of freedom. As such, they cannot be used to perform the non-analytical term correction at the moment. Additionally, the FHI-aims interface does not currently support these corrections, but will in the near future.

**Fig. 8:**
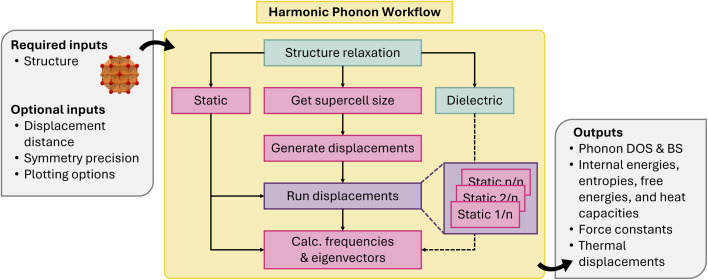
Schematic of harmonic phonon workflow.

The workflow can be described as follows: in the first (optional) step, the structure is fully optimized with a strict force convergence. This is essential to ensure that the forces from the displaced supercells do not contain any spurious noise from residual forces. Next, the supercell transformation is determined based on the minimum length of each lattice vector. The supercell is generated such that it is as cubic as possible, to ensure that the forces converge better with the supercell size. Supercells with displacements are created by phonopy based on the unit cell symmetry (the number of displacements is determined dynamically) and jobs for the computation of the forces created. In the last step, these forces are used by phonopy to compute the force constants and subsequent band structure and density of state plots. Fig. 8 shows a workflow diagram. In addition to the phonon dispersion, the workflow also outputs the phonon density of states and thermodynamic properties such as the heat capacity and free energy.

Similar workflows have been implemented in other frameworks. One noteworthy example is the implementation in AiiDA, which can utilise force calculations from a variety of DFT codes (VASP, Quantum ESPRESSO,^92,93^ FHI-aims, *etc.*). Other implementations are available in the pyiron framework and with the FHI-vibes package.^94^ All of these implementations rely on phonopy for the calculation of the dynamical matrix.

Our implementation of the harmonic phonon workflow has been used in recent studies on MLIPs.^39,95^ Instead of a DFT code, a universal MLIP was employed to calculate the forces for the displaced supercells. The studies serve as a benchmark for the accuracy of MLIPs and show that the workflow can create phonon dispersions from any force Calculator implemented in atomate2. The workflow has been extended to run at different cell volumes so that the thermal expansion and the Grüneisen parameter can be calculated (see below).

#### 4.1.9 Equation of state workflow

The zero-temperature limit of a solid's equation of state (EOS) is a frequently-used tool to study its cohesion and response to compressive and expansive strain. The solid-state EOS typically relates the energy *E* of a solid to its volume *V*, or to its pressure *p*. Both formulations are in essence equivalent because the first law of thermodynamics indicates that *p* = –(*dE*/*dV*)_*S*_ (at constant entropy *S*). Various theoretical models for a “universal” EOS have been developed. Their construction and utility as applied to sp-bonded solids is discussed in ref. 96, and are applied HT to diverse materials in ref. 97. These theoretical models enable one to extrapolate the energy and volume relation beyond those computed directly and extract information such as the solid-state cohesive energy, equilibrium volume, and bulk modulus.

**Fig. 9:**
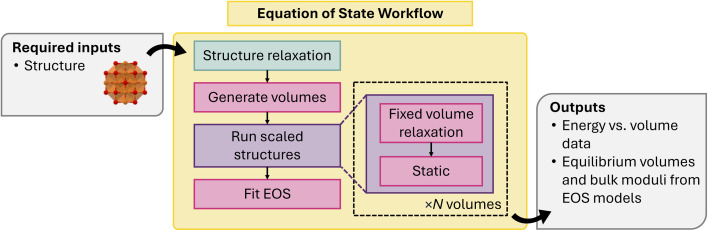
Schematic of equation of state (EOS) workflow.

To generate an EOS, one performs a set of fixed-volume relaxations of a crystal at different volumes. By relaxing the atomic positions within a cell of fixed volume, one is typically better able to fit the resultant energies to a model EOS. The base implementation in atomate2 is abstract: the CommonEosMaker class defines only a workflow for computing a set of energies for an input crystal at different volumes (by default, six volumes within a range of ±5% of the input structure volume). Optionally, the user can relax the structure closer to its equilibrium volume before performing the EOS-specific calculations. The workflow is illustrated in Fig. 9.

Concrete implementations of the EOS workflow exist for VASP, including MP-compliant parameters, FHI-AIMS, and MLIPs. In the MLIP implementation ForceFieldEosMaker, a convenience method called from_force_field_name allows one to generate an EOS workflow solely from the name of an atomate2-supported MLIP and input structure. By default, the EOS data is then fit to a handful of theoretical models from authors including Murnaghan,^98^ Birch,^99^ Poirier and Tarantola,^100^ or Vinet and coworkers.^101^ The extrapolated EOS parameters (minimum energy, equilibrium volume, bulk modulus, *etc.*) are stored in a dictionary for each model EOS alongside the original energy and volume data for later analysis.

#### 4.1.10 Quasi-harmonic approximation workflow

**Fig. 10:**
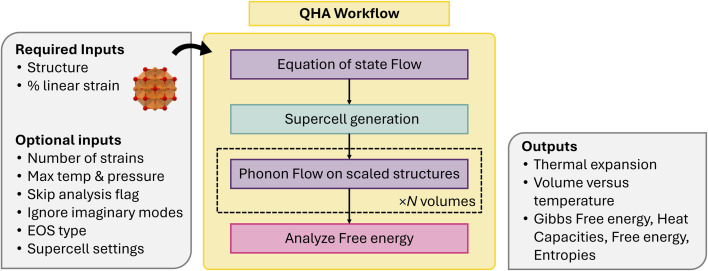
Schematic of quasi-harmonic approximation (QHA) workflow.

To calculate the thermal expansion of compounds at finite temperatures, atomate2 has an implementation of the quasi-harmonic approximation (QHA) workflow,^102^ integrating both phonon and equation-of-state (EOS) workflows. The current QHA workflow relies on phonopy to compute thermal properties, *i.e.*, free energy, for determining the unique minimum value of Gibbs free energy by varying volume. The workflow (Fig. 10) starts with the equation of state workflow to apply linear strain to the structure and relax the structure under the constraint of constant volume. Subsequently, the harmonic phonon workflow based on the finite displacement method is applied to each scaled structure. Based on the temperature (*T*) dependent free energies *F*(*V*, *T*) computed at the different volumes *V* we can evaluate the influence of anharmonicity based on the volume expansion on the thermal properties.

To obtain the free energies, we sum the total DFT energy *E*_0_(*V*) to the vibrational part of the free energy *F*_vib_(*V*; *T*) at different volumes, *V*, according to

1 *F*(*V*, *T*) = *E*_0_(*V*) + *F*_vib_(*V*; *T*).

The current implementation does not consider contributions to the free energy beyond harmonic vibrations and therefore might not be suitable for metals or alloys where electronic or configurational entropy can be non-negligible. Our goal is to incorporate these impacts in future versions. The Gibbs free energy as a function of pressure *p* and temperature *T* is obtained as2
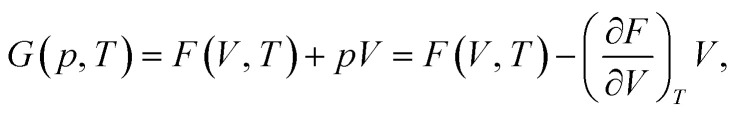
where the pressure is replaced by 
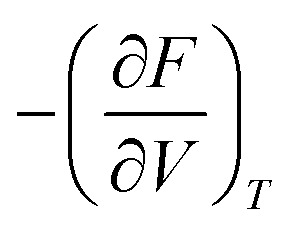
. This equation can be evaluated with the help of an equation of state fit of *F*(*v*) at fixed temperature, similar to the EOS workflow (Section 4.1.9). Currently, implementations for MLIPs and VASP are available, with the goal to expand support to other calculators in the near future.

#### 4.1.11 Mode Grüneisen parameter workflow

The mode Grüneisen parameter (MGP) is a key metric for quantifying the anharmonicity of specific vibrational modes in a crystal. It plays a crucial role in determining lattice thermal conductivity, driving thermal expansion, and enabling phase transitions. atomate2 includes an implementation of the mode Grüneisen parameter workflow, which exclusively relies on the changes in phonon frequencies at different volumes for a given compound.

**Fig. 11:**
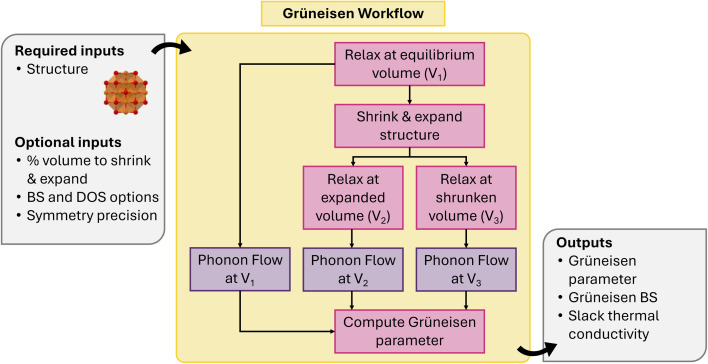
Schematic of mode Grüneisen workflow.

The MGP workflow begins with an initial structural relaxation, followed by two additional relaxations conducted at slightly expanded and slightly compressed volumes (Fig. 11). Subsequently, phonon computations are performed for all three structures using phonopy. With the phonon frequencies of the three structures at hand, the mode Grüneisen parameter *γ*_**q***ν*_ at the wave vector **q** and band *ν* is defined as3
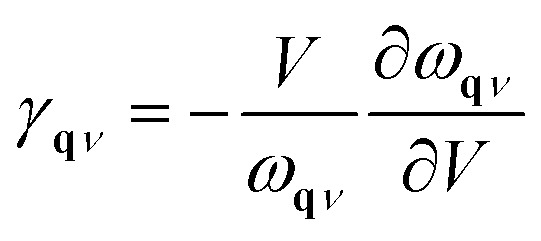
4
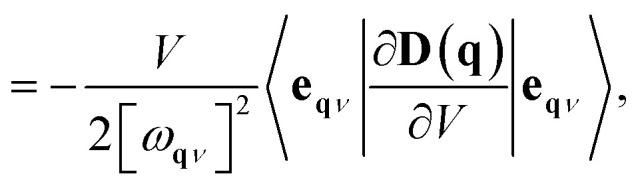
where *V* is the primitive-cell volume, *ω* is the phonon frequency, **D** is the dynamical matrix, and **e** is the eigenvector. The above equation can be approximated using the finite difference method. In our workflow, phonopy is used to compute the mode-dependent Grüneisen parameters on a regular mesh and along a high-symmetry path. The average Grüneisen parameters are obtained following ref. 103 as5
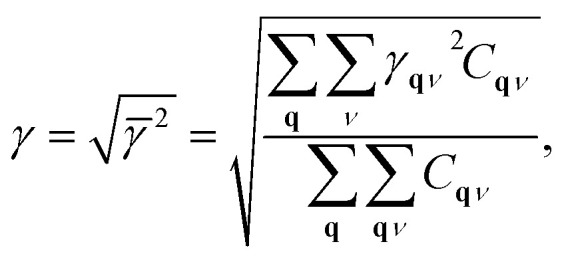
where *C* refers to the mode-specific heat capacity.

#### 4.1.12 Electron phonon band-gap renormalization workflow

The electron–phonon interaction (EPI) is a fundamental determinant of the optical properties of solids. It contributes to the temperature dependence and quantum zero-point renormalization (ZPR) of critical point energies. atomate2 provides a workflow for electron–phonon bandgap renormalization, following the methodology of Zacharias and Giustino^104^ (ZG) and as implemented in VASP.

**Fig. 12:**
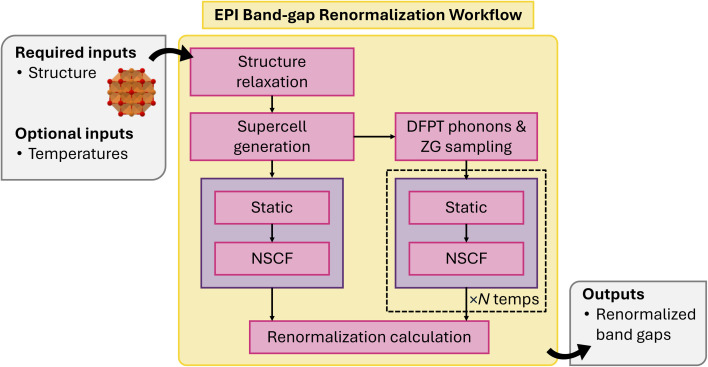
Schematic of electron–phonon band gap renormalization workflow.

The workflow begins with a structural relaxation using tight convergence criteria to eliminate the presence of imaginary phonon modes (Fig. 12). A large supercell (>15 Å) is constructed to provide sufficient convergence of the renormalised properties. Next, the phonon frequencies and eigenvectors are obtained using DFPT. Subsequently, the ZG special displacement approach is employed to construct displaced supercells that yield accurate thermal averages of the mean squared atomic displacements.^104^ We note, an alternative approach is to employ Monte-Carlo sampling of displacements,^105^ however the ZG method enables convergence of properties in a one-shot approach. The displaced supercells are constructed using the implementation available in VASP, with one supercell generated for each temperature of interest. For each structure, a uniform band structure calculation is conducted, comprising a static calculation followed by a uniform non-self-consistent field (NSCF) calculation. Meanwhile, a corresponding band structure calculation is performed on the equilibrium supercell to serve as a reference for calculating the renormalized band gap. Finally, the renormalized band gap at each temperate is obtained by comparing the band gap of the equilibrium structure with the averaged band gap calculated from the temperature-dependent displaced structures.

#### 4.1.13 Anharmonic phonons workflow with hiPhive/Pheasy

Lattice dynamics is a critical field in materials science, describing key thermal properties such as the thermal expansion coefficient, lattice thermal conductivity, and phase stability at various temperatures. Historically, computing these properties accurately and efficiently in a HT mode has been challenging, and a streamlined workflow for LD would significantly advance materials engineering and contribute to computational materials databases. AIMD is one of the more accurate ways to model lattice dynamics, however, it is time-consuming and cost-ineffective. An alternate approach employs perturbation theory and interatomic force constants (IFCs). These are defined by the Taylor expansion of the total energy with respect to atomic displacements.

Second-order IFCs, which define phonons, can be calculated through DFPT, finite-displacements, or the random-displacement method. These calculations allow for the derivation of macroscopic thermal properties at the harmonic level (see Section 4.1.8). Anharmonic IFCs, which are crucial for properties like thermal expansion and lattice thermal conductivity, are more difficult to compute due to combinatorial explosion of terms. Even at the third-order level, high compute efficiency is necessary to achieve wide-scale deployment to small and large systems. Recent advancements in sampling IFCs from high-information-density configurations have made the calculation of anharmonic IFCs more feasible. Tools such as CSLD,^106,107^ ALAMODE,^108^ hiPhive^109,110^ and Pheasy^111^ enable the fitting of IFCs to any desired order with few training samples and have paved the way for HT computing of thermal properties.

**Fig. 13:**
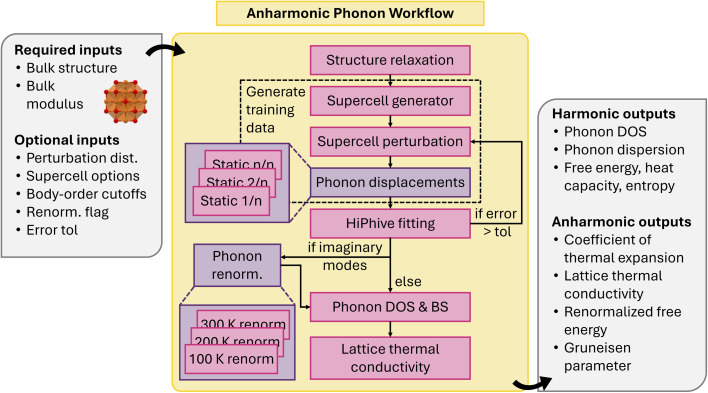
Schematic of anharmonic phonon workflow.

Our anharmonic phonon workflow^112^ automatically calculates interatomic force constants up to 4th order from perturbed training supercells, and uses them to obtain lattice thermal conductivity, coefficient of thermal expansion, and vibrational free energy and entropy. The workflow starts with a primitive structure and adjustable parameters such as the force field Calculator, hiPhive/Pheasy fitting options, and temperatures of interest (Fig. 13). The optimum supercell size is obtained following the same process as the harmonic phonon workflow. A series of random perturbations are performed and the energies and forces obtained using a static calculation. This dataset is subsequently used for fitting force constants using hiPhive or Pheasy. Harmonic phonon properties are calculated using phonopy, while lattice thermal conductivity is obtained using FourPhonon^113^ and phono3py.^89,114^ There is also the option to renormalization the phonon band structure using techniques from thermodynamic integration. The workflow is dynamic: for example, if the fitting RMSE exceeds a certain threshold, the workflow will automatically add a new displacement calculation to increase the training set size, ensuring the accuracy and reliability of the results.

The atomate counterpart of the same workflow has been utilized for calculating lattice dynamical properties from first principles, as detailed in ref. 112. This paper demonstrates the application of the workflow in calculating interatomic force constants, lattice thermal conductivity, thermal expansion, and vibrational free energies. Deployment of either workflow at a large scale would facilitate materials discovery efforts towards functionalities including thermoelectrics, contact materials, ferroelectrics, aerospace components, as well as general phase diagram construction.

#### 4.1.14 MPMorph workflow

While determining the ground-state crystal structure of ordered materials is relatively straightforward, determining the equilibrium structure of disordered materials is challenging. Disordered materials include glasses, amorphous materials, and alloys, and may exhibit different structural motifs at different temperatures and physical conditions. Typically, one must perform NpT-ensemble MD to equilibrate disordered materials. However, NpT-MD is quite expensive, making it less appealing for HT applications such as amorphous material dataset generation.^115^

The MPMorph workflow^80,81^ circumvents NpT equilibration by recursively fitting a set of NVT-MD runs to an equation of state (EOS). After performing the minimum number of fixed-volume calculations needed to fit a standard EOS, the code fits an EOS and determines if the extrapolated equilibrium volume *V*_0_ lies within the range of volumes already computed. If *V*_0_ lies outside this range, the workflow rescales the volume to *V*_0_ and repeats the fitting and analysis steps until *V*_0_ is in range. As a fail-safe, the workflow terminates if an in-range *V*_0_ cannot be determined after a set number of steps. A final NVT “production run” is then performed at volume *V*_0_ with a quench temperature schedule to move the atoms into their lower-temperature disordered configuration. A few different options for the quench temperature schedule are available: a “slow” quench, which ramps down the temperature in NVT, and a “fast” quench, which performs a *T* = 0 DFT relaxation of the structure.

**Fig. 14:**
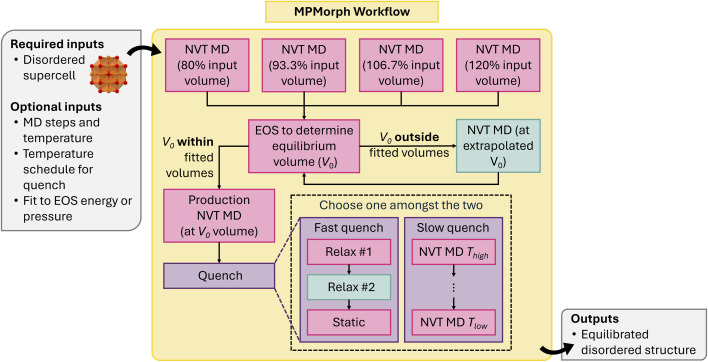
Schematic of MPMorph workflow.

The current MPMorph workflows, visualized in Fig. 14, have been generalized to a code-agnostic framework that only requires the user to define MD jobs for the various stages of the run (initial equilibration, and production). Code-specific implementations for VASP and MLIPs are currently available.

#### 4.1.15 Magnetic ordering workflow

Magnetic materials are of great interest due to their technological applications (*e.g.*, magnetic refrigeration, data storage, spintronic devices) and role/relationship in complex physical properties (*e.g.*, superconductivity, multiferroics, *etc*). However, due to the combinatorial complexity of identifying the ground-state configuration in the magnitude and direction of magnetic spins in a lattice, magnetic orderings are often overlooked in HT DFT studies. Unfortunately, this can sometimes lead to significant impacts on both the calculated ground-state energy and properties of the material (*e.g.*, bandgap), especially for transition metal oxides and other commonly studied materials.^116^

Collinear magnetic spin configurations (*i.e.*, up and down spins) can be modeled through conventional DFT codes, such as VASP. Previously, Horton *et al.*^117^ established a scheme for enumerating the likely collinear magnetic orderings for a given input structure, and, using relaxation and static energy calculations performed with VASP, identifying the ground-state collinear magnetic ordering for a given structure. This methodology was implemented as an atomate workflow and has been adapted for atomate2. Unlike its implementation in atomate, it is now written as a common workflow, allowing it to be easily adapted to other DFT codes.

**Fig. 15:**
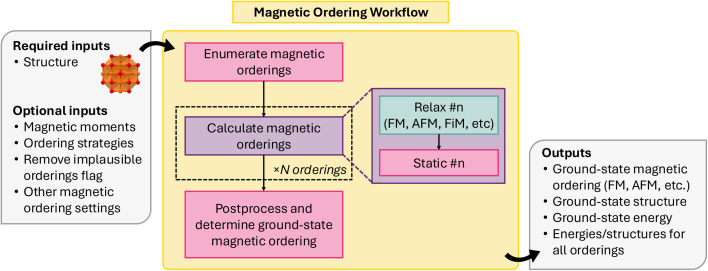
Schematic of collinear magnetic ordering workflow.

Fig. 15 shows a schematic for the collinear magnetic ordering workflow, implemented in atomate2 as MagneticOrderingsMaker. The workflow consists of three overarching jobs: (1) magnetic ordering enumeration, (2) relaxation and energy calculations with DFT, and (3) post-processing to determine the ground-state ordering.

#### 4.1.16 Adsorption workflow

Investigating surface adsorption is a crucial process in understanding electrode behavior and heterogeneous catalysis. Surface adsorption is a complex process involving molecules attaching to a material's top layer, encompassing both physical and chemical reactions. DFT calculations can be utilized in examining preferred surface facets, along with adsorption thermodynamics and kinetics. Due to the complexity of the adsorption process, special attention must be paid to the relaxation of potential adsorption sites.

Montoya and Persson^118^ previously established a streamlined workflow for modeling surface adsorption in atomate. The workflow involves constructing distinct adsorbate configurations for arbitrary surface terminations to efficiently handle the extensive DFT calculations. The workflow in atomate2 retains the core structure of the original workflow while integrating jobflow for enhanced automation. This revised workflow supports the automated generation of symmetrically distinct adsorption sites, calculating the enthalpy energies for adsorbed surface configurations and the reference states, as well as returning the optimized structures and their adsorption energies.

**Fig. 16:**
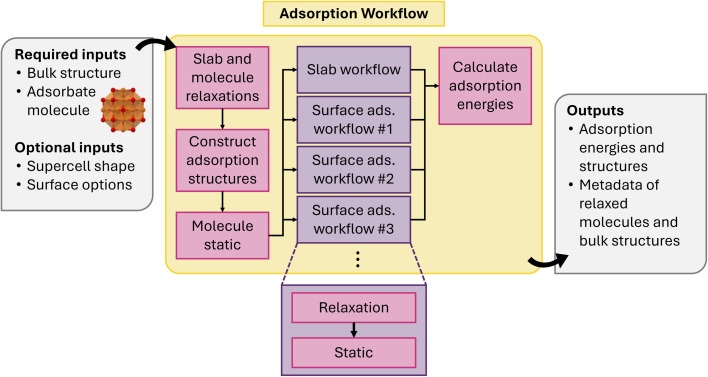
Schematic of adsorption workflow.

The workflow starts from the relaxation of a bulk structure and target molecule (Fig. 16), followed by a static calculation to obtain a reference energy for the molecule. In the second step, the workflow then performs the surface adsorption site searching to generate surface-adsorbate configurations based on the Miller index. The workflow includes default parameters for the thickness of the slab and vacuum, the length and width of the surface, and the surface miller index. For each potential adsorption site, the workflow performs a relaxation followed by a static to obtain the energy. A slab without any adsorbate is generated for the reference state of the surface. Finally, the adsorption energy for surface-adsorbate configurations is calculated by subtracting the enthalpy from the two reference energies of molecules and slab. The outputs of the workflow include the relaxed surface-adsorbate configurations and energies, sorted by ascending adsorption energy. It is possible that during the relaxation calculation, a reaction occurs that decomposes the original molecule structure. At present, the workflow does not include additional analysis to determine whether this has occurred and further validation of adsorption configurations is recommended, especially for complex molecules.

#### 4.1.17 Point defect workflow

The physical properties of semiconductor and optoelectronics materials are often dominated by the presence of point defects in the material.^119,120^ Simulating point defects in an HT manner presents some fundamental challenges that are difficult to overcome and is an active area of research.^121–123^ Defect calculations are typically more computationally expensive due to the need to use large supercells to describe point defects in a dilute limit, as supercells that are too small can suffer from inadequate descriptions of the atomic relaxation around the defect site(s) and their impact on the host electronic structure. Second, simulating charged point defects using a periodic basis set will introduce spurious interactions between the defect and its periodic images, and finite-size corrections are needed to account for this effect.^124,125^ Furthermore, if one is interested in more quantitatively calculating the electronic and optical behavior of defects, more expensive methods like hybrid functionals (*e.g.* HSE06 (ref. 58)) that can describe charge localization better than conventional workhorse exchange-correlation functionals like PBE or SCAN are required to accurately capture the electronic structure of the defect,^126^ leading to even higher computational costs. Additionally, since many defects exhibit nontrivial spin configurations, there is usually no guarantee that the ground-state electronic configuration is achieved, and multiple calculations with different initial conditions might be required.

Addressing all of these challenges simultaneously is not feasible given current computational approaches and resources. As such, we have focused on developing a flexible workflow with two requirements in mind:

(1) The workflow must be modular and allow the user to use any combination of structure and electronic optimizer to obtain the ground energy of a charge defect supercell.

(2) Since we cannot guarantee that the ground-state electronic configuration is achieved, we must design some system to aggregate the results of multiple defect calculations.

This allows the user to perform defect simulations in an HT manner to obtain an initial database of defect properties but also allows users to update the atomic and electronic optimization if lower-energy configurations are found. The composable nature of our workflows and the variety of DFT and MLIPs Calculators supported by atomate2 allows us to thoroughly address (1). Addressing (2) is more challenging, since there is no one-to-one correspondence between the isolated defect you are trying to simulate and the defect supercell used to perform the calculation. There are multiple valid choices for the defect supercell, but they all represent the same isolated defect. To address (2), a structure-based defect object defined using only the unit cell of the host material has been developed, providing a supercell-independent representation of the defect.^127^ This defect object is used as the primary input of the workflow and is also stored alongside each charged-defect supercell calculation to facilitate aggregation of the results from multiple runs of the same defect charge state.

**Fig. 17:**
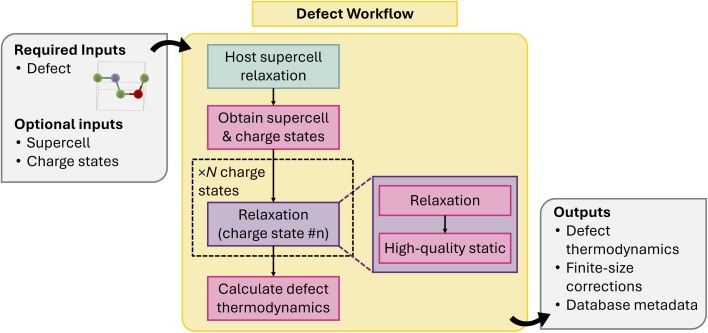
Schematic of point defect workflow.

The defect workflow (Fig. 17) requires the users to first define a supercell relaxation workflow which will take an automatically generated defect supercell and a charge state as input and return the relaxed atomic structure and total energy. By default, these supercell cell relaxations will be composed of a less expensive structure optimization step with a PBE functional, followed by a high-quality HSE06 static calculation. We note that care must be taken with this approach, as local minima for more symmetric and charge-delocalized states favored by PBE may not be able to be overcome by HSE06 calculations initialized with such configurations, which also motivates the need for accessible databasing in (2) for enabling extensible potential energy surface sampling. The full defect workflow will take a defect object as an input and automatically generate the initial defect supercell and determine the possible charge states from formal oxidation states of the species involved in the creating the defect. The supercell relaxation & static workflow will be performed for each charge state and a post-processing step will be called to apply the finite-size corrections and populate, tabulate the energies and other metadata for constructing persistent defect databases.

#### 4.1.18 Anharmonicity quantification workflow

While the harmonic model provides a good approximation to the vibrational frequencies and modes of a material, it is incapable of predicting properties that arise from purely anharmonic effects such as thermal conductivity or the lattice expansion coefficient.^128^ There are multiple approaches to include these effects, ranging from third-order perturbative approaches to fully anharmonic molecular dynamics trajectories, with varying degrees of accuracy and computational cost. Quantifying the level of anharmonicity in a material is therefore necessary to ensure efficient calculations of these materials properties.

The anharmonicity quantification workflow uses the output of the harmonic phonon workflow (Section 4.1.8) as a starting point to calculate the anharmonicity metric *σ*^A^ first introduced by Knoop and coworkers.^128^*σ*^A^ estimates the anharmonicity of a material at a temperature, *T*, by taking the ratio between the root mean square error of the forces calculated by the harmonic model and the standard deviation of the actual forces in a thermodynamic ensemble average, assuming the mean force is zero. It is defined as6
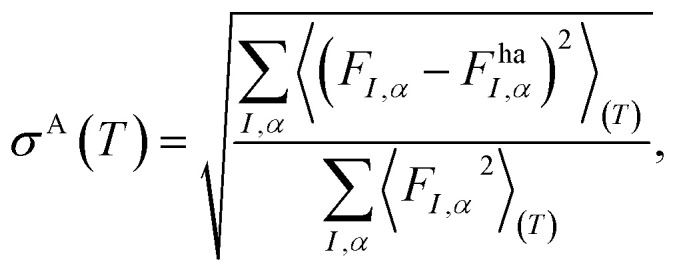
where *F*_*I*,*α*_ is the *α* component of the DFT-calculated forces for the *I*th atom, *F*^ha^_*I*,*α*_ is the same force estimated by the harmonic model, and 〈·〉_(*T*)_ represents a thermodynamic ensemble average at *T*. This metric is widely used in the community, and has been demonstrated to be correlated to properties such as the lattice thermal conductivity.^128^

**Fig. 18:**
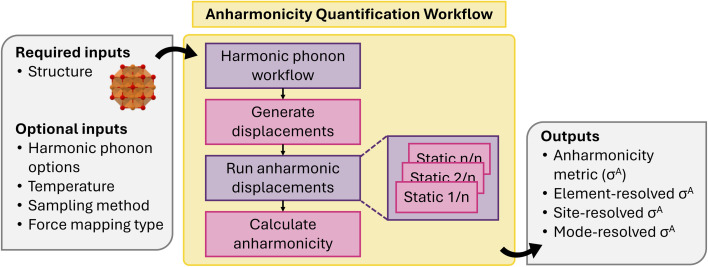
Schematic of anharmonicity quantification workflow.

The workflow to calculate *σ*^A^ is shown in Fig. 18. The first step is to calculate the harmonic force constants using phonopy and the workflow shown in Fig. 8. From here a set of thermally displaced structures are generated by either by harmonic sampling or *via* a one-shot approximation.^94,128^ The one-shot approach approximates the complete thermodynamic ensemble as a single structure, where all atoms are displaced to the classical, harmonic turning points for each vibrational mode.^105^ All generated structures have the same supercell size as the harmonic phonon workflow for consistent results. Next, the forces for each structure are evaluated using the same methodology as the harmonic phonon maker. The sample generation and force evaluations can also be combined with a single molecular dynamics job, but this has not yet been implemented. The calculated forces and displacements are then used to calculate *σ*^A^ for the full structure using eqn (6). The force components can also be masked onto individual element types, lattice sites, or vibrational modes to generate an element-, site-, or mode-resolved *σ*^A^ vector, respectively.

#### 4.1.19 Electrode discovery workflow

Since solid-state batteries can utilize cathode materials that do not contain lithium in the as-synthesized state, the exploration of materials systems through iterative insertion of ions into an atomic structure is an important step in identifying new materials for energy storage applications. Effective intercalation electrodes require “topotactic” ion incorporation where working ions (WIs) are integrated into the atomic structure without major perturbations to the host lattice. Recent studies^129,130^ have demonstrated that analysis of the electronic charge density can reliably predict symmetry-distinct ion insertion sites in the atomic structure which are reliable initial guesses for the ion insertion position. The ion insertion workflow takes advantage of the dynamic workflow generation capabilities of jobflow to iteratively add WIs into an atomic structure based on candidate ion insertion sites identified from the electronic charge density. The output can be aggregated to provide estimates on the voltage profile of the electrode material, which is a key metric for electrochemical performance. Since the workflow only requires standard outputs from any DFT simulation engine, it supports any DFT simulation engine that can compute and store the electronic charge density.

**Fig. 19:**
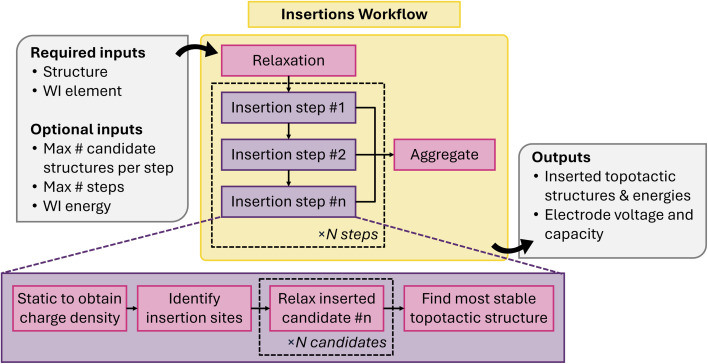
Schematic of electrode insertion workflow.

The electrode workflow (Fig. 19) is composed of a series of repeatable ion-insertion steps that produce the most energetically favorable new structure containing one additional WI. Each ion-insertion step begins with an atomic structure that is topotactically matched to the host lattice or the host lattice itself. The workflow firstly performs a static DFT calculation to obtain the electronic charge density. From the electronic charge density, the workflow identifies symmetry-distinct ion insertion sites and ranks them based on the integrated charge density in a small sphere around the insertion site. For a subset of sites with the least integrated charge density, the workflow performs a DFT structure optimization calculation to obtain the relaxed atomic structure and total energy. Finally, it aggregates these results and filters them to find the lowest energy structure that is topotactically matched to the host lattice, resulting in the input structure for the next ion-insertion step.

The starting structure and the WI species are the only required inputs to the workflow, however, the behavior of the workflow is flexible and can be controlled by additional parameters. The maximum number of insertion steps and the maximum number of distinct sites to consider at each step can be specified by the user. If not specified, the workflow will continue to insert WIs until none of the new structures are topotactically matched to the host lattice. We note that charge balance could also be used to define maximum insertion, however this functionality has not yet been implemented in the workflow. The final output is a voltage profile of the material in question. However, when the workflow is applied to a large number of structures and chemical compositions, this workflow serves as a systematic way to explore lower symmetry configuration spaces. In these cases, the insertion workflow is used to populate a database with a new structure, and relegate the aggregation of topotactically matched structures and the computation of the voltage profile to a separate post-processing step.

#### 4.1.20 Ferroelectric workflow

Ferroelectrics are insulating materials with a nonzero electric polarization switchable by an applied electric field. Ferroelectric materials have been extensively studied using DFT and the modern theory of polarization.^131^ In this framework, the electronic polarization of a periodic crystal is computed from the Kohn–Sham wavefunctions as a Berry phase. This polarization is a multivalued quantity, defined only modulo a quantum of polarization. The quantum of polarization is an integer multiple of a lattice vector, multiplied by the ratio of charge and unit cell volume. In short, the polarization is a lattice.^132^ In practice, only polarization differences are experimentally relevant, and the spontaneous polarization of a crystal is defined as a change in polarization relative to a nonpolar structure. Therefore, in computing the difference in polarization between two structures, one must select polarizations that are on consistent lattice points, sometimes called branches. A standard approach is to compute the polarization of the polar structure, and a nonpolar reference structure from which it is continuously deformable, as well as several linearly-interpolated structures between polar and nonpolar structures. Then, the polarization values from each of these calculations can be adjusted so that they are consistent and belong to the same smooth branch. Upon identification of this common branch, the polarization difference, or spontaneous polarization, is then computed by simply subtracting the polar and nonpolar polarizations. The spontaneous polarization calculated in this way is directly comparable to experiments.^132^

**Fig. 20:**
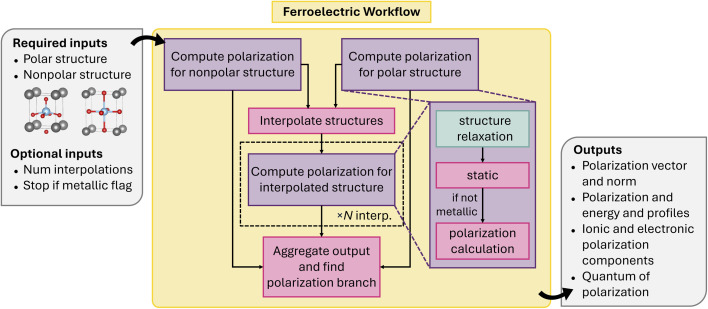
Schematic of ferroelectric workflow.

The present workflow implements this procedure. The workflow inputs are the polar structure of interest and a nonpolar reference structure that is in the same low-symmetry setting of the polar one. In addition, the atoms in both structures have to be in the same order so that the intermediate structures between the nonpolar and polar endpoints can be generated using a linear interpolation. The workflow begins by calculating the polarization of the polar and nonpolar structures (Fig. 20). This includes an optional relaxation followed by a static calculation before the dipole moment is obtained using the Berry phase approach.^132^ In the next stage, the polarization of a number of structures linearly-interpolated between the polar and nonpolar structures are obtained following the same process. Finally, the output of all calculations is collected and the polarization is computed by finding the common branch. Other outputs include the electronic and ionic contribution to the polarization and the quantum of polarization. The workflow is in principle general and any *ab initio* DFT code can be used, however at present only VASP implementation exists. The workflow was originally developed in atomate by Smidt *et al.*^133^ and extensively used to build two databases of candidate ferroelectrics.^133,134^

We note that determining an appropriate nonpolar reference structure for a given polar structure can be obtained by using the PSEUDO tool^135^ in the Bilbao Crystallographic Server (BCS), as recently done to build the ferroelectrics database in ref. 134. Alternatively, if a nonpolar reference is already known, it can be transformed into the polar setting by using the Structure Relations tool in BCS, as was done to build the ferroelectrics database in ref. 133.

#### 4.1.21 Materials Project workflow

The bedrock of the MP database is an extensive library of about 150 000 DFT-relaxed structures with corresponding thermodynamic and electronic properties. To generate these structures, two sets of workflows were developed in atomate to relax an input structure and obtain its total energy: one for PBE and PBE+*U*^59^ and one for r^2^SCAN.^136^ The PBE/+*U* workflow has been used since the inception of MP, whereas the r^2^SCAN workflow was more recently introduced^137^ and used to study the properties of about 33 000 materials in MP.

These workflows have been rewritten in atomate2 with minor modifications to improve their robustness. In both cases, the atomate2 workflows consist of two sequential relaxations followed by a static total energy (single-point) calculation. This is due to a quirk of VASP, wherein electronic properties, such as the density of states, are not physically meaningful after a relaxation, as they are averaged over previous ionic configurations, and do not correspond to the final relaxed structure. To both speed the workflow and potentially stabilize complex calculations, the wave function from a given step is used to initialize the subsequent calculation in the workflow. Note that neither atomate flow used this initialization scheme, and that the atomate r^2^SCAN flow did not perform a final static calculation.

In the PBE/+*U* workflow, two relaxations with PBE/+*U* are performed followed by a PBE static. When a material containing Co, Cr, Fe, Mn, Mo, Ni, V, or W and either O or F is studied, PBE+*U* is automatically used; otherwise PBE without a +*U* is used.^10,138^ In the r^2^SCAN workflow, consistent with ref. 137, a coarser PBEsol relaxation (at a larger force convergence tolerance) is followed by a finer r^2^SCAN relaxation (at a smaller force convergence tolerance), followed by an r^2^SCAN static at its self-consistent relaxed geometry. Thus the outputs of the flows are a structure corresponding to an energy, eigenvalue spectrum, and density of states. This information is then used to build material entries within MP, which include also formation enthalpy (relative to a set of elemental reference configurations) and thus convex hull distance.

The MP input sets are also used to define workflows for determining equations of state (EOS), and other flows. Such a workflow also exists in atomate, and was used in ref. 97. However, the computational demands of this workflow are quite high. For compatibility with ref. 97, a set of “legacy” EOS MP-compatible flows exist in atomate2, along with a set of PBE/+*U* and r^2^SCAN EOS flows which are more tractable in HT, and have recently been used to generate numerous equations of state to aid in benchmarking computational parameters used by the Materials Project.^139^

#### 4.1.22 MatPES workflow

**Fig. 21:**
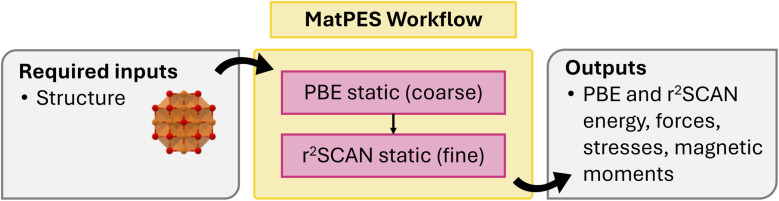
Schematic of MatPES workflow.

The Materials Project potential energy surface (MatPES) workflow (Fig. 21) is designed to strike a good balance between low computational cost and generating high-quality energy, force and stress labels for training foundational MLIPs. It is solely intended to run static calculations at both PBE and r^2^SCAN level of theory where the PBE wavefunction is used as the initial guess to facilitate SCF convergence of the subsequent r^2^SCAN static, significantly reducing the number of electronic steps needed at the more expensive meta-GGA level. This workflow structure also permits multi-fidelity or difference learning between different levels of DFT approximations. The consistent generation of training data across two levels of theory enables systematic comparison of MLIP accuracy with respect to their training data and to experiment. Its development involved carefully tuning VASP convergence settings across chemical systems to ensure high-quality training labels and, most importantly forces.

To date, datasets of 
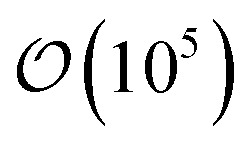
 MatPES calculations have been generated and used to train and/or finetune foundational MLIPs including CHGNet and M3GNet. An initial release of a MatPES-compliant dataset through the Materials Project is forthcoming.

#### 4.1.23 Electronic transport workflow with AMSET

The electronic transport properties of solids determines their use in technological applications, including photovoltaics, thermoelectrics, and power electronics. A wide range of approaches have been developed to model band-like transport in semiconductors, with the linearized Boltzmann transport equation (BTE) being the most commonly used.^140^ Here, a key computational challenge is to accurately obtain the lifetime of charge carriers (electrons or holes) under a range of perturbations (phonons, impurities, grain boundaries, *etc.*). For electron–phonon scattering, the most reliable approach is density-functional perturbation theory often combined with Wannier or Fourier interpolation of matrix elements onto dense *k*- and *q*-point meshes.^141^ Unfortunately, this approach incurs a high computational cost which limits its use to relatively small systems or those with high degrees of symmetry. An alternative approach, termed AMSET,^142^ employs semi-empirical models for the scattering matrix elements based on first-principles inputs. AMSET includes contributions from deformation potential, ionized impurity, piezoelectric, and polar optical phonon scattering. The method provides band and *k*-point resolved insights into the scattering physics of materials. A benchmark on ∼20 compounds revealed an accuracy within 20% of DFPT at three orders of magnitude less computational expense.^142^

The AMSET workflow in atomate2 automates the calculation of all materials properties required to obtain electronic transport. The main inputs are a structure and the temperature and doping concentrations of interest. The workflow begins with an initial structural relaxation with tight convergence settings to avoid the presence of imaginary modes. The elastic tensor, dielectric tensor, piezoelectric tensor, and band structure are obtained using the workflows described above. Deformation potentials are calculated by applying a series of strains to the unit cell, followed by static calculations, and the comparison of the band energies to a reference unperturbed static. The wavefunction coefficients are extracted from a dense band structure calculation, while an averaged polar optical phonon frequency is obtained from the *Γ*-point DFPT calculation used to obtain the ionic dielectric constant. As with all BTE implementations, the resulting transport properties are highly sensitive to the density of the *k*- and *q*-point sampling used to integrate the matrix elements. The workflow includes automated convergence checking to sequentially increase the interpolated mesh density until transport properties converge. Two versions of the workflow are provided, one based on GGA inputs and another more accurate but more expensive version using HSE06. The main outputs of the workflow include the temperature-dependent electronic mobility, conductivity, Seebeck coefficient and electronic contribution to the thermal conductivity. An early version of the workflow was used to obtain the transport properties of 23 000 materials using machine learned materials inputs.^143^

#### 4.1.24 Metal–organic framework workflow

Metal–organic frameworks (MOFs)^144^ are nanoporous materials composed of metal nodes linked by organic linkers. Although MOFs have emerged as leading candidates for applications such as water harvesting, CO_2_ capture, hydrogen storage, and chemical sensing, its community still lacks automated workflows for high-throughput computations.

**Fig. 22:**
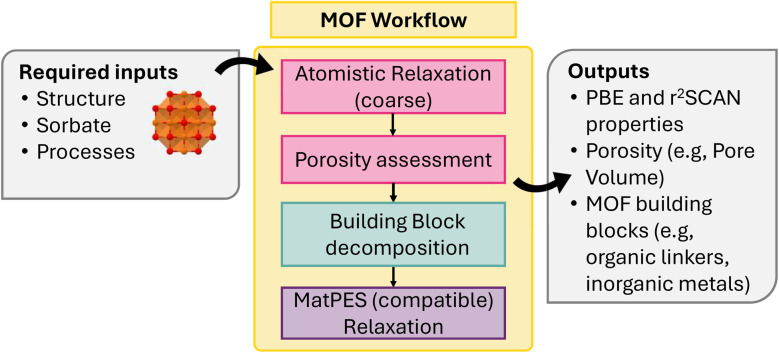
Schematic of the MOF workflow.

In the initial stage of the MOF workflow (Fig. 22), a MOF structure is processed using Zeo++.^145^ The inputs include a crystal structure, a sorbate molecule, and the number of processors for parallelization. Zeo++ extracts various pore characteristics *via* Monte Carlo sampling (*e.g.*, the pore limiting diameter and accessible pore volume), applicable more generally to crystalline porous materials (*e.g.*, MOF, zeolites). The workflow enables users to specify filtering criteria for which a structure is considered porous or meets a given porosity threshold. For example, a candidate structure might be required to (i) exhibit a pore limiting diameter greater than a predetermined value for the given sorbate molecule and (ii) possess a probe-occupiable accessible volume fraction exceeding a set percentage threshold.

For structures far from equilibrium, those passing the initial Zeo++ screening are then subjected to coarse geometry optimization using methods such as MLIPs and semi-empirical DFT. Following this, the workflow permits the user to reapply Zeo++, using similar or novel criteria, for further filtering. The MOF candidates emerging from these steps are then refined using higher-level quantum mechanical computations, analogous to those employed in the MatPES workflow. Where an initial structural relaxation is conducted with PBE-D4, the converged wavefunctions obtained at this stage are used to initialize an r^2^SCAN-D4 relaxation. These wavefunctions are then further employed to compute an r^2^SCAN-D4 single-point calculation at the optimized geometry, yielding well-converged properties such as the phonon and bulk modulus.

Furthermore, an additional module can be plug in using MOFid^146^ that aims to extract individual building block of the MOF (*e.g.*, organic linkers and metal nodes). This functionality may be employed either for post-analysis or as an additional filtering step based on user-defined rules.

### 4.2 Molecular systems

#### 4.2.1 Frequency flattening optimizer workflow

**Fig. 23:**
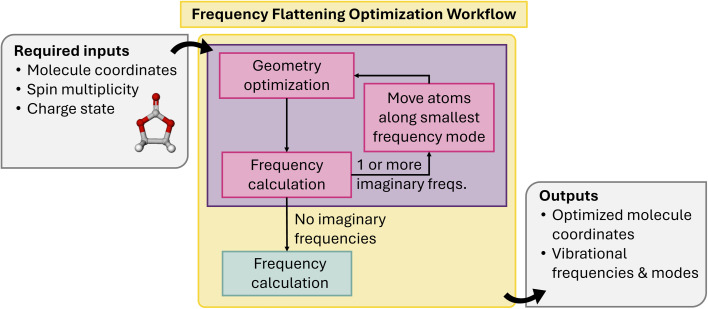
Schematic of frequency flattening optimizer workflow.

Quasi-Newton methods like L-BFGS are the most commonly used iterative optimization methods for geometric optimization in DFT due to their superlinear convergence. Quasi-Newton methods usually approximate the Hessian with a sequence of gradients and steps. This is performed to avoid the computational burden of calculating the exact Hessian at each step, which is the expensive part in most cases. Since the Hessian is approximate, the converged stationary point might be a higher-order saddle point instead of a global minimum on the PES. To guarantee the convergence to global minima on the PES, the frequency flattening optimizer (FFOpt) workflow performs a sequence of geometry optimizations followed by frequency calculations until we reach a global minimum (Fig. 23). Since a higher-order saddle point on the PES is usually characterized by at least one imaginary vibrational mode, examining the output of the frequency calculation provides a straightforward means of assessing whether or not the saddle point geometry is a true minimum on the PES. If it is not, the saddle point geometry is perturbed along the direction of the imaginary vibrational frequency mode, and the optimization is restarted. This process is repeated until all the vibrational frequencies are positive. The workflow allows one negative vibrational frequency of less than 15 cm^−1^ in order to account for numerical noise in the frequency calculation.

## 5 Conclusions and future work

The atomate2 software package incorporates several new features that broaden its capabilities and simplify its use. From a fundamental standpoint, workflows are now built on top of the newly created jobflow library which makes it easier to reuse workflow components and also simplifies data passing between jobs and input/output operations. While not emphasized in the current work, the jobflow library also makes it possible to employ different workflow execution engines, including FireWorks and jobflow-remote, to distribute jobs over computing resources. This change makes it easier to circumvent some issues faced by users of FireWorks, including difficulty running with certain network firewall configurations as detailed in the original FireWorks paper.

The fundamental improvements introduced in atomate2 have facilitated the expansion of the software's scope to encompass a broader array of calculators, including machine-learning based calculators, and to include a larger set of workflows. The framework also allows for workflows that employ a mix of different calculators, which may become more commonplace in the future as MLIPs are used prior to accurate physics-based simulations. A combination of DFT and MLIP calculators within a single workflow has already been used to automatically train MLIPs through random structure searches.^147^ We expect that the capabilities of atomate2 will continue to improve over time. Such potential improvements could simply be the expansion of Calculators and workflows, or may additionally include usability improvements such as calculation dashboards and materials design and submission frameworks. atomate2 is designed to support such community extensions through the python namespace mechanism. As the user community for atomistic and electronic structure calculations grows and calculation methods continue to evolve, software tools must also adapt to meet the changing needs of this community. The improvements implemented in atomate2 represent a path forward to adapting to and accommodating these changes.

By default, atomate2 retains all inputs and outputs of each calculation (unless the user chooses to delete them), thereby preserving complete data provenance. Additionally, because atomate2 currently uses the FireWorks engine to execute workflows, it automatically inherits FireWorks' comprehensive workflow tracking capabilities. All relevant metadata such as job states (waiting, running, completed), timestamps for each state change, and execution details (compute host, return codes, *etc.*), are recorded in a database. This approach ensures that results remain reproducible and that users can verify whether a given calculation has been run before. Moreover, the underlying workflow engine provides advanced control features (*e.g.*, automatic error handling, reruns, and duplicate workflow detection) to prevent redundant computations. In summary, atomate2 offers full workflow provenance tracking and management functionality on par with other state-of-the-art frameworks.

To further support users at all levels, atomate2 provides a suite of educational materials ranging from interactive tutorials and comprehensive documentation to workshop resources and community forums that guide both newcomers and experienced researchers alike. The support resources include:

• Interactive tutorials: a growing collection of hands-on examples is available in the official tutorials repository. These walkthroughs cover fundamental workflows, advanced features, and practical tips, helping users grasp essential concepts step by step. https://github.com/materialsproject/atomate2/tree/main/tutorials.

• Comprehensive documentation: the primary documentation portal, serves as a centralized resource for best practices, API references, configuration details, and troubleshooting guidance. Its modular structure allows users to dive into topics of interest at their own pace. https://materialsproject.github.io/atomate2/.

• Workshop resources: for those seeking more intensive training, materials from the recent CECAM workshop provide in-depth presentations, interactive demos, and hands-on exercises that illustrate real-world scenarios. The workshop page on the CECAM site offers a detailed overview of session topics and supplementary materials. https://www.cecam.org/workshop-details/automated-ab-initio-workflows-with-jobflow-and-atomate2-1276.

https://lhumos.org/collection/0/680bb4d7e4b0f0d2028027ce.

https://lhumos.org/collection/0/680bb4d3e4b0f0d2028027c9.

https://lhumos.org/collection/0/680bb4d0e4b0f0d2028027c5.

https://lhumos.org/collection/0/680bb4c7e4b0f0d2028027c1.

• Community support: new users and veteran practitioners alike can seek help through GitHub issues, and the MatSci public forum, where an active community of developers and collaborators regularly share insights, answer questions, and foster ongoing improvements to atomate2. These combined resources ensure that anyone from newcomers building their first automated *ab initio* workflow to advanced users exploring complex, customized jobs can efficiently get started and receive the support they need to succeed with atomate2.

https://matsci.org/c/atomate/atomat2/55.

https://github.com/materialsproject/atomate2.

## Author contributions

Concept – AG and AJ. Software – HS, KB, TB, JB, AB, JB, XC, YC, JC, OC, CE, MG, JG, SG, REAG, RDG, MH, TJI, AK, RK, MCK, BL, XL, MM, RSM, AN, SP, GP, TARP, FR, BR, JR, GMR, AR, MS, JS, JXS, AS, RS, CT, VT, DW, DW, MW, HY, HZ, JZ, ZZ, AJ. Writing – original draft – HS, KB, TB, JB, AB, XC, YC, JC, OC, CE, MG, JG, SG, REAG, RDG, MH, TJI, AK, RK, MCK, BL, XL, MM, RSM, AN, SP, GP, TARP, FR, BR, JR, GMR, AR, MS, JS, JXS, AS, RS, CT, VT, DW, DW, MW, HY, HZ, JZ, ZZ, AJ. Writing, review, and editing – all authors. Supervision – AG, MA, DC, JG, GH, MH, JN, KP, TARP, GMR, MS, RS, JV, DVF, AJ. Funding – KP and AJ. Project admin – AG and AJ. Methodology – all authors.

## Conflicts of interest

There are no conflicts of interest to declare.

## Data Availability

The code for atomate2 can be found at https://github.com/materialsproject/atomate2 and the documentation for it can be found at https://materialsproject.github.io/atomate2/. The version of the code employed for this study is version 0.0.21. The Zenodo DOI corresponding to the code is https://doi.org/10.5281/zenodo.10677080. The CECAM workshop resources can be found on https://www.cecam.org/workshop-details/automated-ab-initio-workflows-with-jobflow-and-atomate2-1276, https://lhumos.org/collection/0/680bb4d7e4b0f0d2028027ce, https://lhumos.org/collection/0/680bb4d3e4b0f0d2028027c9, https://lhumos.org/collection/0/680bb4d0e4b0f0d2028027c5, https://lhumos.org/collection/0/680bb4c7e4b0f0d2028027c1. The community support for atomate2 can obtained at https://matsci.org/c/atomate/atomat2/55 and *via* the Issues section of the atomate2 GitHub repository. There is no separate dataset to declare.
