# Correction: Atomate2: modular workflows for materials science

**DOI:** 10.1039/d5dd90036k

**Published:** 2025-08-18

**Authors:** Alex M. Ganose, Hrushikesh Sahasrabuddhe, Mark Asta, Kevin Beck, Tathagata Biswas, Alexander Bonkowski, Joana Bustamante, Xin Chen, Yuan Chiang, Daryl C. Chrzan, Jacob Clary, Orion A. Cohen, Christina Ertural, Max C. Gallant, Janine George, Sophie Gerits, Rhys E. A. Goodall, Rishabh D. Guha, Geoffroy Hautier, Matthew Horton, T. J. Inizan, Aaron D. Kaplan, Ryan S. Kingsbury, Matthew C. Kuner, Bryant Li, Xavier Linn, Matthew J. McDermott, Rohith Srinivaas Mohanakrishnan, Aakash A. Naik, Jeffrey B. Neaton, Shehan M. Parmar, Kristin A. Persson, Guido Petretto, Thomas A. R. Purcell, Francesco Ricci, Benjamin Rich, Janosh Riebesell, Gian-Marco Rignanese, Andrew S. Rosen, Matthias Scheffler, Jonathan Schmidt, Jimmy-Xuan Shen, Andrei Sobolev, Ravishankar Sundararaman, Cooper Tezak, Victor Trinquet, Joel B. Varley, Derek Vigil-Fowler, Duo Wang, David Waroquiers, Mingjian Wen, Han Yang, Hui Zheng, Jiongzhi Zheng, Zhuoying Zhu, Anubhav Jain

**Affiliations:** a Department of Chemistry, Imperial College London London W12 0BZ UK a.ganose@imperial.ac.uk; b Department of Materials Science and Engineering, University of California Berkeley California 94720 USA; c Energy Technologies Area, Lawrence Berkeley National Laboratory Berkeley CA 94720 USA ajain@lbl.gov; d Materials Science Division, Lawrence Berkeley National Laboratory Berkeley California 94720 USA; e Program in Applied Mathematics, University of Arizona 617 N. Santa Rita Tucson Arizona 85721 USA; f UCLouvain, Institut de la Matière Condensée et des Nanosciences, Université catholique de Louvain Chemin des Étoiles 8 Louvain-la-Neuve 1348 Belgium; g Institute of Physical Chemistry, RWTH Aachen University 52074 Aachen Germany; h Department Materials Chemistry, Federal Institute for Materials Research and Testing (BAM) Berlin Germany; i National Renewable Energy Laboratory Golden Colorado 80401 USA; j Department of Materials ETH Zürich Zürich CH-8093 Switzerland; k Institute of Condensed Matter Theory and Solid-State Optics, Friedrich Schiller University Jena Jena Germany; l Department of Chemical and Biological Engineering, University of Colorado Boulder Boulder Colorado 80302 USA; m Radical AI, Inc. New York City NY USA; n Thayer School of Engineering, Dartmouth College Hanover NH 03755 USA; o Microsoft Research AI for Science USA; p Department of Civil and Environmental Engineering, The Andlinger Center for Energy and the Environment, Princeton University Princeton NJ USA; q College of Chemistry, University of California Berkeley CA 94720 USA; r Department of Physics, University of California Berkeley CA 94720 USA; s Kavli Energy NanoSciences Institute at Berkeley Berkeley CA USA; t Matgenix SRL, A6K Advanced Engineering Centre Square des Martyrs 1 6000 Charleroi Belgium; u Department of Chemistry and Biochemistry, University of Arizona 1306 East University Boulevard Tucson Arizona 85721 USA; v The NOMAD Laboratory, Fritz-Haber-Institut der Max-Planck-Gesellschaft Faradayweg 4–6 D-14195 Berlin Germany; w Department of Chemistry, University of Colorado Boulder Boulder Colorado 80302 USA; x Cavendish Laboratory, University of Cambridge J. J. Thomson Ave Cambridge UK; y WEL Research Institute Avenue Pasteur 6 1300 Wavre Belgium; z Department of Chemical and Biological Engineering, Princeton University Princeton NJ USA; a Lawrence Livermore National Laboratory Livermore CA 94551 USA; b Molecular Simulations from First Principles – MS1P e.V. Clayallee 167 14195 Berlin Germany; c Department of Materials Science of Engineering, Rensselaer Polytechnic Institute Troy New York USA; d Institute of Fundamental and Frontier Sciences, University of Electronic Science and Technology of China Chengdu 610054 China; e School of Chemistry and Biochemistry, Georgia Institute of Technology Atlanta GA 30332 USA; f Bakar Institute of Digital Materials for the Planet, University of California Berkeley CA 94720 USA

## Abstract

Correction for “Atomate2: modular workflows for materials science” by Alex M. Ganose *et al.*, *Digital Discovery*, 2025, **4**, 1944–1973, https://doi.org/10.1039/D5DD00019J.

There is an error in Aakash Naik name in the author list of the original manuscript. The correct name, as given in the author list of this Correction, is “Aakash A. Naik”.

The Royal Society of Chemistry apologises for these errors and any consequent inconvenience to authors and readers.

